# Exploring Deep Transfer Learning for Medical Image Processing and Analysis: A Comprehensive Analysis across Modalities

**DOI:** 10.2174/0115734056456208260706064206

**Published:** 2026-07-14

**Authors:** M.A. Sithi Banu, A. Dhavapandiammal, Kalavathi Palanisamy

**Affiliations:** 1 Department of Computer Science and Applications, The Gandhigram Rural Institute (Deemed to be University), Dindigul, India

**Keywords:** Medical imaging, Transfer learning, Deep learning, Deep transfer learning, Federated learning, Advanced imaging technologies

## Abstract

In recent years, medical imaging has become an important tool for diagnosing diseases and disorders in healthcare. Advanced imaging technologies are being developed for non-invasive and early detection of diseases and disorders. Analyzing medical images by clinical experts is very expensive. To overcome these challenges, developing automated methods provides an effective solution. Consequently, for processing and analyzing medical images, researchers have adopted the emerging Deep Learning (DL) technologies. It has proven effective across several industries, most notably in healthcare. Even so, it has two significant limitations, such as the training cost and the large amounts of labeled data required. To reduce these limitations, Transfer Learning (TL) and Deep Learning (DL) have been integrated to create Deep Transfer Learning (DTL). This reduces the need to start from scratch and eliminates dependencies by leveraging knowledge from a source task to a target task during training, using fewer datasets. This review addresses the definitions, concepts, modalities, tasks, and techniques of DTL, along with public and private datasets used as source and target data in network-based medical imaging approaches. It also categorizes the last seven years of research by human anatomical area. It offers readers comprehensive coverage of technological advancements, future research directions, and challenges. It also reviews DTL methods by discussing those that have been applied, including Federated Learning (FL) for DL. Empirical evidence from recent studies demonstrates that fine-tuning and network-based DTL strategies, including federated learning, consistently enhance diagnostic accuracy, robustness, and generalization across multiple medical imaging modalities, particularly in data-limited clinical scenarios.

## 1 INTRODUCTION

Medical imaging is a significant discipline in which the internal structures of organs and tissues, as well as the body's components, are visualized for diagnosis and treatment. A number of medical imaging modalities have emerged in recent years, in addition to conventional radiological imaging, which produces high-quality digital images. Many different types of medical imaging systems have been developed from simple and common radiological treatments such as X-rays, Computed Tomography (CT), Mammography, Radiography, Ultrasound (US), and Magnetic Resonance Imaging (MRI). Positron Emission Tomography (PET) and Single Photon Emission Computed Tomography (SPECT) measurements are now available on any nuclear medicine equipment. In recent years, rapid advances in hospitals and diagnostic centers have enabled the capture and processing of vast amounts of images at a whole new scale. Diagnostic imaging techniques help diagnose and plan treatments for a wide range of diseases, like cancer, kidney disease, psychiatric disorders, hepatic and biliary pathology, diseases related to cognitive deficit, fractures, and tumor detection [1]. These kinds of images are essential to the area of medical image processing, particularly in DL, a branch of artificial intelligence that deals with nonlinear datasets.

Deep Learning (DL) has successfully tackled several challenging applications over the past few years, especially those involving non-linear datasets. Recent developments in DL techniques have enabled many applications across a wide range of fields, including numerical data analysis and prediction, image processing, voice recognition, and Natural Language Processing (NLP) [2]. Before the advent of DL, Transfer Learning (TL) was referred to as domain adaptation and focused on relating homogeneous data sets using Machine Learning (ML) algorithms [3, 4]. In many real-world scenarios, though, this presumption is not always true. Therefore, conventional machine learning algorithms typically face three major challenges, such as a lack of data, limited computational capacity, and distributional incompatibility. First, many solutions, including distributed learning, cloud computing, data synthesis, and data augmentation, have been proposed to address the first two issues, but each has drawbacks, including security, cost, and efficiency [5]. TL was recently brought to our notice to address all three of these challenges.

The analysis of medical images is still confronted with severe challenges, including the scarcity of labeled clinical data, the variability of domains across imaging modalities and institutions, and the extreme computational complexity of DL models. Most current studies focus on task-specific implementations, thereby lacking an analytic perspective on the performance of DTL strategies in segmentation, classification, and detection. Concurrently, there is a significant need for a structured and comprehensive analysis framework for DTL techniques, network designs, and learning strategies in medical imaging. Traditionally, medical imaging modalities such as X-ray, ultrasound, Computed Tomography (CT), Magnetic Resonance Imaging (MRI), and Positron Emission Tomography (PET) relied heavily on visual assessment and manual interpretation by clinicians. Early Computer-Aided Diagnosis (CAD) systems were primarily based on handcrafted features and conventional machine learning algorithms, which limited their diagnostic performance and generalizability across different imaging modalities. With the evolution of DL, analysis of medical imaging has focused on data-driven feature learning, enabling more precise, automated detection, segmentation, and classification.

DTL has played a major role in advancing medical image analysis in recent years. It enables the transfer of knowledge from large natural and medical image datasets to clinical applications with limited annotated data. This way of working helps a model to converge faster, generalize better, and yield better diagnostic performance. Nowadays, the implementation of AI has turned the mode of medical imaging practice upside down. AI-powered systems assist doctors by automatically interpreting images, facilitating consistency among observers, and delivering rapid and dependable diagnostic support. In particular, DL models have demonstrated remarkable effectiveness in disease detection, segmentation, and classification, thereby improving diagnostic accuracy, increasing clinical efficiency, and enhancing patient care. AI in medical imaging has revolutionized healthcare by enabling large-scale screening programs, personalized treatment planning, and early disease detection, among others. As a result, the interpretation of medical images has shifted from a mainly manual to an intelligent, data-driven process to assist clinical decision-making.

Among the various AI techniques, Transfer Learning (TL) has emerged as an effective strategy for overcoming the challenges associated with limited annotated medical data. When implemented using deep neural networks, TL is commonly referred to as Deep Transfer Learning (DTL). DTL leverages knowledge acquired from a source domain or task to enhance performance on a related target task, thereby reducing the need to train models from scratch using large datasets [5–7]. This approach significantly decreases training time, computational costs, and data annotation requirements, making it particularly suitable for medical imaging applications. Furthermore, DTL helps mitigate distribution mismatches between datasets, addresses the scarcity of labeled training samples, and improves model generalization by transferring meaningful feature representations from related domains [5]. Ongoing advancements in DTL continue to drive the development of more robust and adaptive AI systems through progressive learning strategies. As a result, DTL has become an increasingly important research area in artificial intelligence and machine learning, with widespread applications across diverse domains [8].

DL models have achieved remarkable success across various domains; however, their performance often depends on the availability of large-scale, high-quality training datasets. Acquiring such data can be challenging and expensive, particularly in sensitive fields such as healthcare and financial services, where privacy regulations restrict data sharing. Moreover, traditional centralized training approaches require data to be collected and stored in a single location, raising concerns about privacy, security, and computational costs. Federated Learning (FL), first introduced by Google in 2016, addresses these limitations by enabling collaborative model training across decentralized data sources without transferring raw data to a central server [9]. Although FL preserves data privacy, the performance of federated models may be affected by data heterogeneity and domain shifts among participating clients. To overcome these challenges, DTL can be incorporated into the federated framework to transfer knowledge from data-rich domains to data-scarce domains, improving model generalization and convergence. This integration, known as Federated Transfer Learning (FTL), combines the privacy-preserving advantages of FL with the knowledge-sharing capabilities of DTL, thereby enhancing learning performance across clients with diverse and non-identically distributed datasets.

### 1.1 Deep Learning

Deep Learning (DL) is a part of AI and ML that works on nonlinear data sets and is at the forefront of technology in sectors such as cybersecurity, visual recognition, text analytics, and, especially, healthcare. Developing a suitable DL model is, however, a difficult task due to its changing nature and the absence of a fundamental understanding, turning it into a black box that makes it difficult for ordinary developers to use [10]. DL techniques can handle enormous training datasets by acquiring a concise yet rich visual representation and thereby improving performance. Deep Neural Networks (DNNs) are composed of stacked layers of nodes with activation functions and weights. They can be partially or fully connected and trained using back-propagation and optimization algorithms. Since 2011, Convolutional Neural Network (CNN) layers have enhanced DL models in image tasks. At present, the majority of DLs employ CNN layers that learn directly from the input without requiring human feature extraction, making them more powerful than conventional networks. CNNs are widely used in visual recognition, NLP, medical image analysis, and many other applications because they are specifically designed to deal with a variety of 2D shapes. Several CNN variations are available in the field, such as Xception, ResNet, AlexNet, Inception, and Visual Geometry Group (VGG), where these can be applied in different domains based on their capacity for learning [8, 11].

### 1.2 Transfer Learning

Transfer Learning (TL), a machine learning approach that extends the knowledge gained from working on one task to other related tasks, has delivered outstanding results in a wide range of situations. Previously, AI models were trained from scratch for each task, which meant that large amounts of labeled data and extensive computational resources were necessary. TL allows for knowledge to be transferred from one model to another, thus cutting down on the size of training datasets and accelerating learning. It has been proven to be very effective in different fields such as computer vision, NLP, healthcare, and autonomous systems [12]. In the field of computer vision, pre-trained models on large datasets like ImageNet are further trained for specific tasks to detect objects, scenes, and features in new domains more quickly and with higher accuracy [13]. When the target dataset has very little labeled data, TL can reuse image representations from a large dataset for generic object identification to help prevent overfitting; however, the dependence on data bias can limit its value [14].

ImageNet is a WordNet-based large-scale picture ontology that aims to populate 80,000 synsets with millions of annotated images structured according to a semantic hierarchy. TL is crucial in healthcare because it allows models trained on various medical datasets to rapidly adapt to new tasks, including analysis of medical imaging and discovery of medication [15]. These models can swiftly adapt to newly acquired datasets by leveraging information from a single medical domain, enabling more precise, faster healthcare solutions [16]. Figure **1** illustrates the concept of transfer learning.

#### 1.2.1 History of Transfer Learning

The concept of TL in neural networks was introduced in the 1970s and has since become the basis for modern methods. The discriminability-based transfer is an innovative method that transcends the limitations of traditional transfer-based techniques and accelerates learning in networks launched by DBT, which is why it is essential in current machine learning processes [17]. TL has now become a significant part of contemporary machine learning, enabling practitioners to create reliable models with less data and fewer resources [6]. Domain adaptation was the focus of significant work in the early 2000s. Domain adaptation is a sub-area of transfer learning that addresses how a model trained in one domain can be transferred to another. The role of deep learning in transfer learning in the visual recognition task has been described previously [18]. It discusses deep convolutional networks trained on one task that can later be used for other tasks with sparse data. Deep learning has greatly facilitated transfer learning, especially through very deep convolutional neural networks (ConvNets). One of the primary reasons for the upsurge in transfer learning, thanks to deep learning, is the availability of models trained on large-scale datasets such as ImageNet, showing the power of deep image features to enhance performance in a variety of image recognition tasks and benchmark datasets [19]. Since its theoretical foundations were established in the 1990s, TL has evolved into a fundamental component of modern machine learning. The integration of DL mechanisms has further enhanced the efficiency and effectiveness of TL by enabling the transfer of learned representations across diverse applications. As a result, TL continues to play a crucial role in advancing artificial intelligence, driving innovation across numerous domains and remaining an important area of research. Figure **2** illustrates the historical evolution of transfer learning.

The remainder of this study is organized as follows. The DTL section presents the fundamental concepts, categories, and approaches of DTL. The Methodology section describes the procedures and criteria adopted for this review. Applications of DTL in medical image processing and analysis are discussed in the corresponding section. The Challenges and Opportunities section highlights the major limitations, emerging trends, and future directions of DTL in medical imaging. Finally, the Conclusion section summarizes the key findings and contributions of this review.

## 2 DEEP TRANSFER LEARNING

TL has emerged as an effective approach for addressing machine learning problems where obtaining large amounts of labeled data is difficult or costly. While most DL models require extensive annotated datasets for training, such datasets are often unavailable in many real-world applications, particularly in medical imaging. TL alleviates this limitation by transferring knowledge acquired from a source task or domain to a related target task, thereby reducing the need for large labeled datasets. DTL extends the principles of TL to Deep Neural Networks (DNNs) by leveraging knowledge learned from pre-trained deep models. Unlike conventional machine learning approaches, which often require training models from scratch, DTL leverages pre-trained networks and fine-tunes them for new tasks. This adaptation can involve retraining the entire network or updating only selected layers, depending on the similarity between the source and target tasks.

DTL can be understood from two complementary perspectives, namely problem-level categorization and solution-level categorization. Problem-level categorization focuses on the relationship between the source and target domains or tasks. This relationship is generally characterized by (i) the availability of labeled data and (ii) the degree of domain similarity. Based on label availability, TL can be categorized into inductive, transductive, and unsupervised transfer learning. Additional paradigms, such as cross-modality transfer learning and negative transfer, have also been investigated [5]. Furthermore, based on domain characteristics, TL can be classified as homogeneous transfer learning, where the source and target domains share similar feature spaces, or heterogeneous transfer learning, where the domains differ significantly in their feature representations. Figure **3** illustrates this conceptual taxonomy and highlights the nature of TL problems independent of the underlying DL implementation.

In contrast, solution-level categorization describes how knowledge transfer is achieved within DL models [10]. From this perspective, TL approaches can be broadly classified into instance-based, feature-based, model-based, and relational or adversarial methods. These categories differ in their mechanisms for transferring knowledge, including the reuse of source-domain samples, the learning of domain-invariant feature representations, the transfer of model parameters, and the alignment of feature distributions across domains. Together, these approaches provide a comprehensive framework for implementing TL in DL applications.

In recent years, network-based approaches have become the dominant paradigm in Deep Transfer Learning, particularly in medical imaging applications. These approaches leverage pre-trained deep neural networks and adapt them to target tasks through techniques such as fine-tuning, layer freezing, progressive learning, and the incorporation of task-specific layers [8]. Network (model)-based approaches constitute the core of DTL and are among the most widely adopted strategies in medical imaging applications. These approaches facilitate knowledge transfer and domain adaptation between source and target datasets by leveraging pre-trained deep neural networks and modifying their architectures or parameters to suit new tasks [20]. More recently, FL has emerged as a privacy-preserving extension of network-based DTL, allowing the model to collaboratively train on distributed datasets without exchanging raw data. Figure **4** presents a systematic overview of implementation-level DTL techniques, with particular emphasis on network (model)-based and adversarial-based approaches. While Figure **3** illustrates the conceptual taxonomy of TL problems, Fig. (**4**) highlights the corresponding DL implementation strategies, thereby distinguishing the general TL framework from DTL-specific methodologies. The major strategies employed within the network-based approach are described as follows:

### 2.1 Fine-tuning

Fine-tuning adapts a pre-trained model to a target task by initializing the network's parameters with those learned from a source domain. Depending on the similarity between the source and target tasks, either the entire network or selected layers can be retrained using target-domain data. Due to its simplicity and effectiveness, fine-tuning remains the most widely used DTL technique. It reduces training time, lowers computational costs, and minimizes the need for large annotated target datasets.

### 2.2 Freezing CNN Layers

In this approach, selected layers of a pre-trained Convolutional Neural Network (CNN) are frozen, meaning their parameters remain unchanged during training. Only the remaining layers, typically the fully connected layers, are updated using target-domain data. This strategy preserves previously learned feature representations while reducing the computational burden of retraining.

### 2.3 Progressive Learning

It extends a pre-trained model by preserving existing knowledge and incrementally learning new tasks by adding new network layers. This concept was introduced by Google DeepMind in 2016 through Progressive Neural Networks (PNNs). Similar to human learning, progressive learning builds new capabilities on top of previously acquired knowledge. To prevent catastrophic forgetting, the parameters of pre-trained networks are frozen while newly added layers are trained on target-domain data [21, 22].

### 2.4 Federated Learning

An ML technique that enables model training across distributed data sources. It is among the current DL Trends because it addresses privacy concerns and is well-suited to distributed data settings.

Unlike traditional centralized learning approaches, FL enables model training directly on local devices or institutional servers without requiring the sharing of raw data. Instead, only model updates or learned parameters are exchanged and aggregated to construct a global model. This privacy-preserving framework aligns with the growing demand for secure and decentralized DL systems. In healthcare, FL facilitates collaboration among hospitals and research institutions by enabling the development of predictive models while maintaining the confidentiality of sensitive patient data. Furthermore, Federated Transfer Learning (FTL) extends this paradigm by combining the benefits of TL and federated learning, thereby enabling knowledge sharing across distributed and heterogeneous datasets. Consequently, FTL has gained increasing attention in privacy-sensitive domains such as healthcare and finance. Another important category of DTL is Adversarial-Based Learning, which aims to learn transferable, domain-invariant feature representations from both source and target domains. By minimizing discrepancies between domain distributions, adversarial methods improve knowledge transfer and enhance model generalization across different tasks and datasets.

Among the various DTL techniques, fine-tuning remains one of the most widely used and effective approaches. The popularity of fine-tuning stems from its simplicity, flexibility, and effectiveness in improving model performance. In addition, it reduces training time, lowers computational costs, and minimizes the requirement for large annotated target datasets, making it particularly suitable for medical image analysis and other data-constrained applications.

Furthermore, it has been used with tabular (numerical) and imaging datasets. DL models are composed of layers that extract features, gather information, process data, and perform classification. In an image-related framework, CNN layers remove edges, corners, and small patches, recognize objects or faces, and then classify them using fully connected lateral layers. However, additional research in this field employs complex, combinational techniques, such as ensembles and weighting schemes, to address TL in DL [3]. To the best of our knowledge, however, a search for recent developments in DTL for real-world applications yields techniques that are primarily network (model)-based and have few adversarial components. Figure **3** defines the TL problem type, whereas Fig. (**4**) describes the DL strategies used to realize transfer in practice.

## 3 METHODOLOGY

A systematic literature search was conducted across major academic databases, including IEEE Xplore, ScienceDirect, Web of Science, PubMed, Scopus, and Google Scholar, to provide a comprehensive review of current DTL approaches for medical imaging tasks such as segmentation, classification, and detection. The search was limited to English-language publications within the last seven years, with a concentration on peer-reviewed journal articles, reviews, and conference proceedings. Various studies were identified by combining the relevant key terms, including medical imaging, segmentation, classification, detection, and DTL. An initial screening of abstracts and titles was conducted to determine relevance, followed by full-text evaluations of selected articles. The criteria for including publications required that the studies use DTL algorithms to analyze medical imaging across different modalities, such as X-ray, ultrasound, computed tomography, magnetic resonance imaging, and others, for at least one of the three core tasks. Peer-reviewed sources, literature on other fields, and redundant records were filtered out. Bibliographies of the chosen manuscripts were then examined in order to find some other related research. All contributions mentioned were reviewed and classified by task type, imaging modality, model architecture, and learning strategy, making it easier to synthesize the current trends, strengths, and limitations of the field.

To ensure reproducibility, predefined Boolean search queries were used across the participating databases. The key search query that was used was “DTL” OR TL OR Domain Adaptation AND “Medical Imaging” OR MRI OR CT OR X-ray OR ultrasound AND Segmentation OR Classification OR Detection. Database filtering was then applied to narrow down the retrieved results. The first search provided approximately 520 records in all databases. Following the elimination of duplicate articles, 410 articles were subjected to title and abstract screening, during which 230 were discarded as irrelevant. In the end, 180 full-text articles were screened for eligibility, and 80 were excluded for failing to meet the inclusion criteria. Finally, 100 studies were retained for qualitative synthesis in this review.

The selection process of the overall study is summarized as a PRISMA-style flow diagram, which illustrates the identification, screening, eligibility, and inclusion stages (Fig. **5**).

## 4 DEEP TRANSFER LEARNING IN MEDICAL IMAGE PROCESSING AND ANALYSIS

The main goal of medical image analysis is to identify the anatomical regions affected by the illness, so doctors can better understand how abnormalities develop. TL is a good method for training DNNs, namely DTL, using a small dataset for medical analysis [23]. DTL is a fast-growing field in medical image processing that leverages pre-trained DL models to tackle challenging applications, such as segmentation, classification, and disease detection in healthcare, as shown in Fig. (**6**). It may help overcome the need for a large dataset by applying the network weights or learned parameters of highly trained CNN models to a large dataset (such as ImageNet) to address issues in processing medical images. To accomplish this, the fully connected layers of a well-trained CNN model are trained from scratch using the medical dataset. In contrast, the convolutional layers are either frozen or fine-tuned for feature extraction [24]. TL emphasizes capturing low-level characteristics in medical datasets, thereby minimizing the need for large datasets, memory usage, and training time by employing two TL approaches, such as feature extraction and fine-tuning [25]. A survey of medical imaging analysis [26] reports that only a few DTL studies were identified in the literature up to 2017. Thus, the major goal of the current review was to compile research on medical image processing and analysis that applied DTL techniques to ImageNet and other datasets. We focused specifically on DTL to examine research characteristics across various medical image tasks, such as segmentation, classification, and disease detection. Medical imaging tasks based on DTL approaches are discussed in the succeeding section.

### 4.1 Methods of Medical Image Segmentation using DTL

Medical image segmentation is an advanced field that radically transforms the way medical problems are addressed and diagnosed. The process includes the accurate detection and elimination of some objects or sites in medical images, such as MRI, CT, and X-ray images. The specifics of DTL methods for medical image segmentation are provided in the following explanation:

Xu *et al*. (2017) [27] employed TL to improve brain MRI segmentation by leveraging a VGG network pre-trained on the ImageNet dataset. By leveraging knowledge from large-scale natural image datasets, the proposed approach significantly improved segmentation performance, particularly in scenarios with limited training data. This capability is especially valuable in medical imaging, where annotated datasets are often scarce. Similarly, Xu *et al*. (2018) [28] incorporated TL into a Fully Convolutional Network (FCN) based on a pre-trained VGG architecture. Using modified FLAIR images, the method achieved accurate segmentation of white matter hyperintensities by effectively transferring feature representations learned from ImageNet. The study demonstrated the effectiveness of TL in detecting and segmenting subtle anatomical structures while reducing the need for extensive annotated datasets.

Oliveira and Santos (2018) [29] proposed a TL-based framework for anatomical structure segmentation in chest radiographs. Built upon an unpaired image translation architecture, the method was designed for unsupervised and semi-supervised settings where labeled data are limited. Experimental results demonstrated effective segmentation of anatomical structures, including the clavicle and heart, highlighting the potential of TL in challenging medical imaging scenarios. Mozaffari and Lee (2019) [30] investigated domain adaptation through TL for the automatic extraction of tongue contours from ultrasound images. Their study examined different adaptation strategies within the U-Net architecture and demonstrated that fine-tuning the entire decoder significantly improved segmentation accuracy and generalization across datasets.

Amin *et al*. (2019) [31] utilized pre-trained AlexNet and GoogleNet models combined with TL and hybrid CNN-clustering techniques for brain tumor segmentation. Their approach improved segmentation accuracy by exploiting knowledge learned from large-scale datasets. According to Chen *et al*. (2019) [32], employed the Med3D framework and a two-stage segmentation network for liver segmentation, achieving improved accuracy through TL and shape refinement. Soudani and Barhoumi (2019) [33] applied TL using ImageNet-pretrained VGG16 and ResNet50 models to classify and segment dermoscopic images into five categories. Their results demonstrated improved segmentation performance compared with conventional approaches.

Puybareau *et al*. (2019) [34] proposed a TL-based method for left atrial cavity segmentation using a pre-trained VGG16 network. By employing a pseudo-3D strategy that processes 2D slices while preserving volumetric information, the method achieved accurate segmentation of cardiac MRI data. Lee and Nishikawa (2020) [35] explored both conventional TL and cross-organ cross-modality transfer learning (XTL) for breast lesion segmentation in mammography and MRI. Their findings indicated that XTL outperformed traditional TL by achieving greater overlap between predicted and ground-truth segmentations, demonstrating its potential for image-guided therapeutic applications. Nazi and Abir (2020) [36] combined TL with the U-Net architecture to improve skin lesion segmentation. Likewise, Vu *et al*. (2020) [37] developed a modified U-Net incorporating an ImageNet-pretrained VGG16 encoder, enabling accurate segmentation of thoracic organs at risk, including the heart, lungs, esophagus, and spinal cord. Vidal *et al*. (2021) [38] proposed a two-stage TL framework in which pre-trained weights were preserved through layer freezing. Knowledge learned from MRI-based segmentation models was transferred to chest X-ray analysis, resulting in substantial improvements in lung segmentation performance despite limited training samples.

Cheng and Lam (2021) [39] demonstrated that combining TL with data augmentation improved rib segmentation in lung ultrasound images. Their study evaluated the V-Unet and X-Unet architectures and found that domain-specific pretraining strategies improved segmentation accuracy. In contrast, Karimi *et al*. (2021) [40] investigated the impact of TL on multiple medical segmentation tasks and reported that while TL accelerated model convergence, its effect on final segmentation accuracy depended strongly on the similarity between source and target domains.

Liu *et al*. (2021) [41] introduced a two-stage cross-domain TL framework based on CoVSegNet for COVID-19 lung infection segmentation in CT images. By integrating data-level and model-level TL, the method achieved superior performance compared with existing segmentation approaches. Hsiao *et al*. (2021) [42] applied TL for total kidney volume estimation and demonstrated that a lightweight ResNet50-based encoder significantly improved segmentation performance.

Ankenbrand *et al*. (2021) [43] utilized TL with models pre-trained on 1.5T and 3T MRI datasets, resulting in improved segmentation accuracy and better generalization across different imaging conditions. Zhu *et al*. (2022) [44] proposed the FS-Net framework for automated kidney tumor classification and segmentation in 3D CT images. By leveraging TL from large auxiliary datasets, FS-Net achieved superior performance compared with conventional fine-tuning approaches.

Araujo *et al*. (2022) [45] developed a melanoma segmentation framework using TL, fine-tuning strategies, and DL architectures, including U-Net and LinkNet. Among the evaluated models, U-Net achieved the highest segmentation accuracy. Maiello *et al*. (2022) [46] employed TL to improve lung CT segmentation and demonstrated its suitability for clinical applications. Their results showed enhanced segmentation quality and more accurate estimation of lung volumes and aeration compartments, with performance varying across different disease conditions, including COPD, ARDS, and COVID-19.

The research by Ambesange *et al*. (2022) [47] describes a Federated Transfer Learning (FL) method for organ segmentation that leverages TL across datasets of varying sizes. The strategy also uses local healthcare data to enhance model performance. Lung segmentation accuracy is improved using pre-trained weights from a U-Net segmentation model. The Explainable AI method is utilized to explain the results, which show near-perfect segmentation accuracy with various techniques. A pre-trained MobileNetV2 model is integrated as the encoder in a UNET architecture that uses TL by Riaz *et al*. (2023) [48]. The model can precisely segment malignant lesions using the TL approach, thereby greatly speeding up training. This is important for the early diagnosis of lung cancer. The model was adjusted, proving that TL successfully modified the previously trained model to fit the particular task of lung tumor segmentation.

In a study by Boyd *et al*. (2023) [49], stepwise TL enabled expert-level automated pediatric brain tumor auto-segmentation and volumetric assessment. This method may facilitate the development and translation of AI-based image segmentation algorithms for data-sparse settings. Utilizing Inception V3, SeResNet50, VGG19, DenseNet121, InceptionResNetV2, and EfficientNetB0 as the backbone for the U-Net topology to accomplish the segmentation task, Sharma *et al*. (2023) [50] proposed a U-Net model for segmentation and compared it with six pre-trained TL models. Several TL models were assessed by Ali and Agrawal (2024) [51], including a hybrid model based on a CNN-enabled ResNet101 trained with the VGG-19 stack. In brain tumor segmentation, this model outperforms all previous methods.

Several methods for brain tumor segmentation achieved better results, including pre- and post-processing strategies, and Ren *et al*. (2024) [52] developed a 3D U-Net model based on TL. Srivastava *et al*. (2024) [53] proposed a novel method for analyzing brain tumors using 3D UNets, custom CNN models, and pre-trained TL models (VGG16, ResNet50, InceptionV3, MobileNetV2, and DenseNet121). This method has proven to be state-of-the-art in segmentation problems. The application of TL to improve the TrailMap model's axon segmentation performance in light-sheet microscopy pictures is investigated by Oostrom *et al*. (2024) [54]. This contrasts the effectiveness of the original TrailMap model with that of a refined version that uses expert-annotated noradrenergic axonal structural data. This comparison seeks to highlight the effectiveness of TL in improving model correctness.

Basha *et al*. (2024) [55] proposed an efficient framework for brain tumor diagnosis that combines TL, Gradient-weighted Class Activation Mapping (Grad-CAM), and Mask R-CNN-based segmentation. By leveraging TL and region-based feature extraction, the proposed model demonstrated robust performance in brain tumor detection and segmentation. Clark *et al*. (2024) [56] reported that TL improved segmentation performance across multiple Organs At Risk (OARs), including the spinal cord, optic nerves, submandibular glands, brainstem, brain, parotid glands, lenses, and mandible. Their findings highlighted the effectiveness of TL in enhancing segmentation accuracy, particularly in institutions with limited training datasets.

Several recent studies have further demonstrated the importance of feature extraction strategies within DTL frameworks for medical image segmentation. For example, an enhanced U-Net architecture incorporating attention mechanisms [57] achieved significant improvements in feature representation and Dice similarity scores for lung nodule segmentation in CT images compared with conventional U-Net models. These results emphasize the value of attention-guided feature extraction in accurately delineating small and complex anatomical structures, particularly when training data are limited. A summary of representative DTL-based segmentation techniques and their advantages and limitations in medical image processing is presented in Table **1**.

### 4.2 Medical Image Classification based on DTL Techniques

Medical image classification is a fundamental task in digital image analysis and plays an important role in disease diagnosis, lesion localization, volume estimation, treatment planning, and disease monitoring. Various DTL techniques have been developed to improve classification performance in medical imaging applications.

Samala *et al*. (2017) [58] compared a multitask learning framework with a conventional single-task TL approach for classifying benign and malignant breast masses in mammograms. Their results showed that multitask supervised learning provided superior generalization to unseen cases and demonstrated significant potential for extending lesion classification to digital breast tomosynthesis applications. Meng *et al*. (2017) [59] introduced a liver fibrosis classification framework for ultrasound images using TL and Fully Connected Networks (FCNet). By combining deep feature extraction with FCNet, the proposed method achieved higher predictive accuracy than existing approaches.

Li *et al*. (2018) [60] employed TL to improve classification performance in situations with limited training data, particularly for Autism Spectrum Disorder (ASD) diagnosis. A Stacked Sparse Autoencoder (SSAE) was first trained using healthy functional connectivity patterns, and the learned representations were subsequently transferred to a Deep Transfer Learning Neural Network (DTL-NN). The results demonstrated substantial improvements in classification accuracy, highlighting the value of TL in neuroscience applications where large datasets are often unavailable.

Based on Byra *et al*. (2019) [61], TL with a pre-trained VGG19 model and fine-tuning strategies improved breast mass classification. The study showed that combining TL with a dedicated color-conversion layer produced superior classification performance compared with conventional approaches. Similarly, Ahmed *et al*. (2019) [62] applied ImageNet-pretrained VGG16 models to ultrasound image classification. Through TL and data augmentation, the model achieved higher accuracy, reduced overfitting, and improved generalization performance. Samala *et al*. (2019) [63] further demonstrated the benefits of multi-stage TL, showing that pretraining CNNs on related auxiliary domains can mitigate challenges posed by limited target-domain data. Their findings also indicated that classification performance depends on both the relative sizes of the source and target datasets and the selected TL strategy.

Swati *et al*. (2019a) [64] proposed a brain tumor classification framework based on TL and block-wise fine-tuning. The approach utilized a pre-trained CNN as a feature extractor while selectively fine-tuning network blocks to improve classification performance. The methodology demonstrated potential for extension to other imaging modalities, including PET, CT, and X-ray images.

Talo *et al*. (2019) [65] developed a DTL framework based on ResNet34 for the automatic classification of normal and abnormal brain MRI scans. The model incorporated data augmentation, learning rate optimization, and fine-tuning, resulting in highly accurate classification performance. Likewise, Deepak and Ameer (2019) [66] employed a pre-trained GoogLeNet architecture to extract discriminative features from brain MRI images. These features were subsequently classified using established machine learning classifiers, achieving superior performance compared with existing methods. Yamada *et al*. (2019) [67] demonstrated that TL with pre-trained CNNs achieved classification performance comparable to that of experienced abdominal radiologists for primary liver cancer diagnosis using Dynamic Contrast-enhanced CT images (DCE-CT). Their findings also indicated that TL improved robustness against image misregistration.

Loey *et al*. (2020) [68] introduced a COVID-19 detection framework that combined DTL with Generative Adversarial Networks (GANs) using chest X-ray images. Among the evaluated architectures, including GoogLeNet, AlexNet, and ResNet18, GoogLeNet achieved the highest classification accuracy while maintaining lower computational complexity. Mehrotra *et al*. (2020) [69] investigated several pre-trained CNN architectures for brain tumor classification in MRI images and reported that AlexNet delivered the best performance among the evaluated models. Rehman *et al*. (2020) [70] applied TL using three widely adopted CNN architectures, GoogLeNet, VGGNet, and AlexNet, for brain tumor classification. Both fine-tuning and feature-freezing strategies were evaluated to extract discriminative visual features. Experimental results demonstrated that the fine-tuned VGG16 model achieved the highest classification accuracy, confirming the effectiveness of TL for medical image classification tasks.

TL-enabled deep CNNs (DCNNs) have been developed by Kaur and Gandhi (2020) [71]. Investigating the potential of several pre-trained DCNN models with TL for the categorization of diseased brain images. ResNet50, AlexNet, VGG-16, GoogLeNet, VGG-19, ResNet101, Inceptionv3, and InceptionResNetV2 were among the pre-trained DCNNs that were employed. The AlexNet model was the most accurate and had the best characteristics among the models assessed. Two classification models that use TL to differentiate between blood microscopic images of leukemia-affected and leukemia-free subjects were proposed by Loey *et al*. (2020) [72]. Both models are used with AlexNet, a pre-trained CNN, and show dominance over the first model on several performance criteria. Rodrigues *et al*. (2020) [73] used several CNN architectures, such as VGG, DenseNet, and ResNet, as feature extractors. These models are optimized for the particular purpose of classifying skin lesions and demonstrate how TL can result in considerable increases in diagnostic precision. The study of Sun *et al*. (2020) [74] introduces a DL approach that uses global labels to classify liver cancer histopathology images. To compensate for the lack of thorough cancer-area annotations, patch characteristics are extracted. Image-level features are obtained by combining multiple-instance learning with TL. The method achieves high accuracy in abnormal or normal classification by resolving large-scale image processing issues and addressing sample insufficiency in liver cancer histopathology images.

The investigation by Gu *et al*. (2020) [75] describes two strategies for detecting cross-domain skin diseases. The first uses a supervised deep convolutional neural network classifier pre-trained on ImageNet and fine-tuned on two skin disease datasets. The second method employs adversarial learning as a domain adaptation strategy to enhance recognition performance. The model's generalization performance is evaluated on two skin image datasets for melanoma identification, cancer diagnosis, and cross-modality learning. The trials demonstrate the method's efficacy in tackling the domain shift problem. The use of TL in breast cancer diagnosis with US images, as detailed in a study by Ayana *et al*. (2021) [76], emphasizes its importance in medical image analysis due to the difficulty of obtaining large training datasets. The findings show that TL regularly outperforms traditional methods by leveraging pre-trained models to improve diagnostic accuracy. The most widely used models are VGG19, VGG16, AlexNet, and InceptionV3, with ImageNet as the primary pre-training dataset. A feature-extraction strategy may be more effective than fine-tuning with limited datasets.

Gour and Khanna (2021) [77] stated that TL is critical for improving the efficiency, effectiveness, and adaptability of CNN models used in medical image categorization, especially for ophthalmology. It allows CNN architectures to be fine-tuned for specific applications, such as detecting ocular disorders in fundus images, using models like VGG16, InceptionV3, ResNet, and MobileNet. The method is beneficial in multi-label settings when multiple diseases may be present. The study shows that fine-tuning produces better results, especially with the VGG16 architecture, as evidenced by the high accuracy. The suggested approach of Aftab *et al*. (2021) [78] makes use of the Apache Spark BigDL library to identify leukemia from microscopic pictures of human blood cells using deep TL with the Convolutional Neural Network (CNN) architecture, GoogleNet. The system effectively detects four forms of leukemia, namely normal, Acute Lymphocytic Leukemia (ALL), Chronic Myeloid Leukemia (CML), Acute Myeloid Leukemia (AML), and Chronic Lymphocytic Leukemia (CLL). The model outperformed the Keras model, achieving 97.33% and 94.78% accuracy in training and validation, respectively.

According to Aswathi *et al*. [79] (2021), InceptionV3 employs TL using pre-trained DL architectures learned on ImageNet. The results demonstrated that the TL models had varying accuracies across classes, highlighting the challenges of distinguishing subtler differences in Diabetic Retinopathy (DR) intensity levels. Using the VGG-16 pre-trained CNN model, El Houby (2024) [98] used the TL approach to aid in diagnosing diabetic retinopathy and classifying its grade levels. Islam *et al*. (2022) [80] classified the X-ray samples into three groups, namely normal, COVID-19, and pneumonia, using a CNN with deep TL and RNN. Features were extracted from four well-known CNN networks and used by the RNN to distinguish among various classes. The VGG19-RNN is considered the most effective network for identifying COVID-19. Lee and Aqil (2022) [81] demonstrated the widespread usage of TL approaches to improve the precision of renal illness classification. By integrating various pre-existing models, a high-accuracy model was created that highlights the value of combining deep TL approaches, with precision on the ResNet101V2 model.

TL helps the hybrid CNN learn more robust features, which are crucial for correctly identifying different skin diseases. It also helps the model generalize. By integrating TL with additional tactics such as segmentation and augmentation, Hasan *et al*. (2022) [82] developed a strategy that improved overall classification performance. Khaled *et al*. (2022) [83] provided a solution to the categorization issue in pneumonia on chest X-ray images, as it is now one of the main causes of death in children. Using five image recognition models trained on over 14 million photos of real things, it employs a technique called TL. The study points out that by using a single TB branch of the picture dataset that Li and Paul developed, TL enables a considerable improvement in the *a priori* prediction. When supervised and unsupervised softmax classifiers were compared, the findings showed that CNN models with RF, SVM, and Naive Bayes classifiers performed better than the others.

Li *et al*. (2023) [84] developed a gradient-weighted class activation mapping (Grad-GAM) technique to improve the model's interpretability and a TL technique to adjust CNN models that have already been trained on natural image datasets to medical image tasks if there is not enough training data. The findings demonstrate that the multimodal neural network for glaucoma (GMNNnet) performs very well with multimodal data and can more effectively display the high-dimensional information of glaucoma. The main use of TL in Namachivayam and Puviarasan (2023) [85] was for feature extraction. To extract convolutional features from brain images and reduce the risk of overfitting, pre-trained models such as Inceptionv3 and VGG19 were used. These models performed better at categorizing brain disorders like Parkinson's, Alzheimer's, and tumors.

Ayana *et al*. (2023) [86] have introduced a vision-transformer-based TL method for classifying breast mammograms. It is found that vision-transformer-based TL outperforms CNN-based TL for breast mammography image categorization, achieving better results with lower computational complexity. To classify kidney cancers, Majid *et al*. (2023) [87] used two fine-tuned TL models within the framework of ResNet-101 and DenseNet-121. The performance of these models was rigorously assessed using several metrics and compared with cutting-edge approaches. The results indicated that the fine-tuned TL models were superior, with DenseNet-121 obtaining excellent accuracy. In a study by Raza *et al*. (2023) [88], the DenseNet with TL was applied to segmented GM slices, with the last two blocks retrained, yielding accurate results in multi-class AD classification.

A CNN that uses TL was developed by Kumar *et al*. (2023) [89] and accurately identifies brain tumors in MRI images, showing considerable improvement over many pre-trained networks, including U-Net, AlexNet, ResNet, and VGG-16. In Islam *et al*. (2023) [90], four TL approaches for brain tumor classification using InceptionV3, VGG19, DenseNet121, and MobileNet were experimentally evaluated. Due to its high accuracy, InceptionV3 outperforms all other models. Multiple TL models (EfficientNetB5, MobileNetV2, DenseNet121, ResNet50, and Xception) are used in Alshahrani *et al*. (2023) [91] to identify and categorize bone marrow cells into seven groups. Compared with the other five refined models, DenseNet121 performed better. Abdulsahib *et al*. (2023) [92] offer a unique TL method for classifying liver cancer from medical images using TL models (VGG-16, ResNet-50, and MobileNetV2). The suggested approach effectively identified liver cancer. Additionally, it states that TL-based models outperform conventional methods and DL models that do not use TL for data classification.

CNN is one of the primary algorithms used in deep TL to classify two crucial liver lesion types, hemangioma and hepatocellular carcinoma (HCC). To categorize the kinds with the highest accuracy, Elbashir *et al*. (2023) [93] use a strategy based on ensembles of the best-selected deep transfer models from the commercial models—VGG16, VGG19, DenseNet, Inception, InceptionResNet, ResNet, and EfficientNet. Using the Advanced Al-Biruni Earth Radius (ABER) optimization algorithm, Alhussan *et al*. (2023) [94] developed a unique approach to enhance breast cancer categorization. CNN optimization, feature extraction using AlexNet based on TL, and data augmentation make up this process. The recommended technique is assessed and achieved with a mediocre outcome. Comparing the obtained results to those of existing methods, the application of TL and an optimized CNN improved classification accuracy.

Classification of Disease Stages in Thamizhvani (2024) [95] explains that different TL algorithms use the 3D segmented images as input after segmentation to classify different stages of Alzheimer's disease (AD), including Cognitively Normal (CN), Early Mild Cognitive Impairment (EMCI), Late Mild Cognitive Impairment (LMCI), and Alzheimer's Disease (AD). By adding TL approaches such as Inception V3, GoogleNet, AlexNet, and VGG16, classification accuracy increases. Zaabi *et al*. (2024) [96] established how TL significantly improves CNN model performance and how well knowledge from trained models can be used. Combining TL and Transformer models, which perform better than standard CNNs, covers the use of Vision Transformers (ViTs) in conjunction with TL, which allows for effective feature extraction and categorization. This strategy shortens training time and eliminates the need for large labeled datasets by focusing on specific brain regions, such as the hippocampus, thereby improving classification performance when using TL with transformers.

Two methods proposed by Mehmood *et al*. (2024) [97] for utilizing Transfer Learning (TL) have demonstrated high diagnostic accuracy for Moderate Cognitive Impairment (MCI) and Alzheimer's Disease (AD). Their models, which were fine-tuned, included the Deep Siamese Network (DN) and the VGG-16 network, each with specific parameters for classifying MCI and AD. This enhances the model's performance and saves time. Including data-augmentation methods also increases TL efficiency, as they can expand the size of the training data. Houby (2024) [98] proposed a framework that uses TL and multiple pre-trained CNNs, such as VGG19 and EfficientNetB0, in the preprocessing and classification phases. The framework proved useful for detecting early COVID-19 from chest X-ray images, demonstrating that TL significantly improves model accuracy in distinguishing COVID-19 images.

According to Mammeri *et al*. (2024) [99], applying TL can significantly improve nodule classification accuracy, thereby enhancing the comprehensive lung cancer detection procedure. YOLO v7 was first tried for both detection and classification, then switched to VGG16, which was pre-trained on the ImageNet dataset; nonetheless, because of the limited amount of data. This method improves categorization performance by leveraging existing knowledge. The model successfully classified nodules and showed fewer misclassifications during testing, indicating that the TL technique performed well. The possibility of a semi-supervised classification model based on contrastive learning for the detection of renal WSIs, including glomeruli, and a TL model was also developed by Liu *et al*. (2024) [100] based on pretraining on mouse glomerulus images, achieving high classification accuracy. The suggested TL model was compared against a semi-supervised learning model and a supervised learning model that shared the same classification backbone and dataset. Based solely on mouse glomerulus images and a limited number of IgA nephritis glomerulus images, the results demonstrate that the supervised learning model's classification accuracy was surpassed by the TL approach.

Legouis *et al*. (2024) [101] present kidney-specific Cell-Level Information ExtractoR (K-CLIER), a TL framework for analyzing kidney illness, using single-cell RNA sequencing (scRNAseq) data. This method enables the evaluation of cell types and states in bulk RNA sequencing data from kidney biopsies. The work emphasizes the significance of modified proximal tubular cells in chronic kidney disease, demonstrating how TL can enhance understanding of the disease's mechanisms and potentially lead to improved diagnostic tools. By utilizing the TL concept, Rajput *et al*. (2024) [102] developed an effective decision assistance system. The three most popular CNN models, VGG19, ResNet-50, and Inception-V3, are used in the framework to extract features using a generic ImageNet dataset. A variety of classification criteria are used to compare their performance with the most advanced CNN models, including ResNet-50, Inception-V3, and VGG19.

Through the utilization of pre-trained models in Singh and Lobiyal (2024) [103], TL is used for improving the classification of brain tumors. This can greatly increase accuracy and efficiency. To categorize distinct types of brain tumors, it uses several DL models, including InceptionV3, Xception, VGG19, and DenseNet201. It emphasizes that, as compared to traditional methods, DL models, especially those based on TL, achieved better performance metrics. The goal of Khaliki and Başarslan (2024) [104] is to classify brain malignancies such as meningioma, glioma, and pituitary tumors based on MR scans. CNN and CNN-based TL models were trained, including VGG19, EfficientNetB4, and InceptionV3. EfficientNetB4 achieved the highest accuracy.

TL system architecture is used by Raina *et al*. (2024) [105] to categorize medical images of Brain MRIs and Chest X-rays, detecting disorders including Glioma, Meningioma, and Pituitary Tumor in MRIs, and Pneumonia in X-rays, while also identifying normal scans. The VGG-16 model was trained on the ImageNet dataset and has demonstrated transformative potential for improving diagnostic accuracy and efficiency. According to Al-Otaibi *et al*. (2024) [106], TL is essential for retrieving rich-level spatial data from MRI images, and the CVG-Net method was proposed. The model employs pre-trained networks such as VGG16. The performance of the models used to classify brain cancers is greatly enhanced by the newly created feature set. The diagnostic framework improves overall efficacy through integration *via* TL, advancing automated brain tumor classification.

Mathivanan *et al*. (2024) [107] illustrate the ability of deep TL architectures to transform the field of brain tumor diagnosis. The use of TL techniques for MRI-based categorization of brain cancers is examined. This assesses the performance of four distinct TL models, such as MobileNetV3, DenseNet169, VGG19, and ResNet152. Among the evaluated models, ResNet152 performed the best. To address the inherent challenges of classifying brain tumors, Wang (2024) [108] developed TL and CNN, an automated brain tumor classification system based on the Efficient-Net B3 architecture. This model shows a notable improvement in accuracy. According to Remya *et al*. (2024) [109], applying TL to pre-trained models improves performance in skin lesion detection tasks and speeds up convergence during training. By integrating multiple imaging modalities, the framework improves the model's diagnostic accuracy. TL and multimodal data analysis are combined, and the classification accuracy of differentiating between skin lesion categories is significantly improved.

The research study by Khalad *et al*. (2024) [110] presents a unique method for improving breast cancer detection in mammograms by combining ensemble DL and progressive TL. A specialized Convolutional Neural Network (CNN) oversees a multi-step diagnosis procedure and the INbreast dataset. The technique makes a substantial contribution to medical image classification by addressing class imbalance and achieving an F1 score of 99% in the final classification of 12 breast cancer types. Devi *et al*. (2024) [111] present a TL strategy for identifying breast cancer utilizing a federated learning model. This model addresses data privacy and the limited availability of labeled data in collaborative healthcare settings. The model's accuracy and generalization improve, but domain shift remains a challenge.

In another article, Albalawi *et al*. 2024 [112] built and tested a federated learning-based convolutional neural network, which is used to classify brain tumors on MRI scans. The model achieved high precision and recall across all tumor types, with an overall accuracy of 98%. The higher performance is due to the federated learning paradigm, which enhances model generalization and information privacy compared to traditional DL frameworks.

In addition to conventional classification tasks, feature extraction–based DTL frameworks have been extended to prognostic analysis in brain tumor imaging. A DL-integrated approach combining volumetric convolutional networks for tumor segmentation with a Replicator neural network has been proposed to extract discriminative radiomic features for glioma survival prediction from MRI scans. This study demonstrates the capability of TL–based feature extraction to support both classification and downstream clinical outcome prediction, thereby enhancing the clinical relevance of DTL in neuroimaging. An overview of the many DTL classification techniques, together with their pros and cons for medical image processing, is given in Table **2**.

### 4.3 Disease Detection in Medical Imaging using DTL Approaches

To detect illnesses or health conditions in individuals who do not exhibit any signs of disease, screening tests are the main way to identify diseases earlier. Lifestyle changes or monitoring are the objectives to lower the risk of illnesses or to identify them early enough to provide the best possible treatment. In medical image processing, the following are some important current detection techniques that use DTL:

In conjunction with a TL method, a fully convolutional network variant of AlexNet (FCN-AlexNet) was used by Yap *et al*. (2018) [113]. This model was modified for semantic segmentation, which is essential for reliably recognizing lesions in US images. This TL technique is contrasted with other algorithms, demonstrating its versatility and effectiveness in addressing the special features of breast US images. Deep TL models, including AlexNet, ResNet18, SqueezeNet, GoogleNet, VGG16, and VGG19, were employed in Khalifa *et al*. (2019) [114] to diagnose Diabetic Retinopathy (DR). According to the overall testing accuracy and performance criteria, the AlexNet model reduced the computational complexity and training time while achieving the best testing accuracy.

The TL-based approach for diseased brain detection that significantly decreased the time needed to retrain the AlexNet was presented by Lu *et al*. (2019) [115]. According to the experimental results, the technique outperformed the state of the art in terms of accuracy. Xiaopeng *et al*. (2020) [116] state that TL has improved the CNNs' ability to identify Juvenile Myoclonic Epilepsy (JME) by using pre-trained models. The Inception_resnet_v2 model correctly diagnosed JME, surpassing other models such as Inception_v3 and Inception_v4. It indicates that integrating diffusion MRI with CNN-based TL is a viable strategy for detecting JME, suggesting its potential to enhance diagnostic assessments. To develop a hybrid model for identifying diabetic retinopathy, Gangwar and Ravi (2021) [117] implemented TL on pre-trained Inception-ResNet-v2 and added a custom block of CNN layers to achieve higher accuracy than an existing technique.

An essential method proposed by Harsono *et al*. (2022) [118] has been shown to improve model performance in the challenging domain of medical image analysis. The study applied TL to the Inflated Inception 3D (I3D) model, pre-trained on a large dataset (ImageNet) and modified for lung nodule identification. This technique shortens training periods while improving performance. The model can detect and classify lung nodules more accurately by using pre-trained weights rather than training from scratch. Multiple well-known DL models, including VGG16, ResNet50, and Inception v3, were used in Islam *et al*. (2022) [119] together with TL methods to identify kidney diseases such as cysts, stones, and tumors on CT scans. TL enables models to leverage pre-trained weights from large datasets, helping them effectively extract features from CT scans and thereby improving classification accuracy.

In a study by Jabbar *et al*. (2022) [120], a framework for the automatic identification and diabetic retinopathy classification using the TL-based VGGNet architecture was developed, and the results showed that the system beat advanced methods in terms of accuracy. The CNN and TL models were optimized to achieve high success and low loss rates, and hyperparameters were updated by Altun *et al*. (2023) [121]. TL models (MobileNetV3-s, ResNET50, EfficientNetV2, VGG19, and DenseNet121) with CNNs were modified; in particular, the MobileNetV3-s model achieved extremely high accuracy in distinguishing between comparable and monkeypox skin lesions.

A modified generative adversarial network technique and multiround TL have been proposed by Chui *et al*. (2023) [122] in response to the research limitations of current lung cancer detection models in multiround TL, negative transfer, and the absence of a bridge between source and destination domains, which greatly improve lung cancer detection performance. A modified Xception model (modified Extreme Inception) by Bhattacharjee *et al*. (2023) [123] primarily consists of transferring the weights of the “ImageNet” to the target network *via* TL, followed by fine-tuning the network for automatic lung cancer and CKD diagnosis. This can differentiate between benign, normal, and malignant lung CT images. Additionally, it can efficiently identify renal CT images into multiple groups, including normal, tumor, cyst, and stone.

The influence of TL on medical object detection was quantitatively examined in Terzi and Azginoglu (2023) [124] using natural and medical image datasets. This TL technique used five alternative weight initialization approaches. Mask R-CNN was chosen as a DL architecture. The results reveal that TL from the medical image dataset successfully detected the tumor, and in-domain TL outperforms cross-domain TL. As Pandey and Bhandari (2024) [125] stated, TL is critical in improving the efficacy and efficiency of DL models for brain tumor detection. Various YOLOv5 models found that using TL improved performance metrics, resulting in higher brain tumor detection rates and greater model robustness, allowing it to adapt to different datasets and improve generalization, which are critical in medical applications.

A VGG-16 TL model is recommended by Ashafuddula and Islam (2024) [126] to aid in the early clinical identification of brain cancers and to develop a ContourTL-Net approach for identifying and categorizing brain tumors using contour detection. The given methodology has shown better results and has great potential to considerably improve the quality and efficiency of brain tumor detection. Table **3** outlines the statistical significance and inherent limitations of many transfer-learning DL methods used in medical image processing.

In addition to conventional CNN-based detection models, recent studies have explored feature extraction–oriented hybrid TL frameworks for brain tumor detection. The XcepFusion model [127] uses a pre-trained Xception backbone, with selective layer freezing and pruning, to extract discriminative deep features while reducing computational complexity. By fusing features from multiple network depths, the framework improves detection robustness and generalization across heterogeneous brain MRI datasets, highlighting the effectiveness of feature extraction and pruning strategies in detection-oriented DTL.

Modern studies have moved past conventional convolutional neural network pipelines with more sophisticated feature space representations as microstructural invariant representations of magnetic resonance imaging of bone and neurodiagnostic studies, and the expanding scope of DL technologies in cardiac magnetic resonance imaging. These studies highlight the growing significance of highly structured representations and modality-selective feature learning, which can be further sharpened through the integration of advanced transfer-learning models.

Overall, a comparative analysis of the reviewed studies indicates that performance improvements are primarily influenced by the choice of backbone architecture, TL strategy, and dataset characteristics, rather than the chronological development of models. Across multiple studies, architectures such as ResNet and DenseNet demonstrate more consistent performance compared to earlier VGG-based models, particularly in handling complex medical imaging tasks.

### 4.4 Comparative Results and Discussion of Deep Transfer Learning Strategies across Medical Imaging Tasks

This section synthesizes the empirical results reported across the reviewed studies and provides a comparative discussion of DTL strategies with respect to task type, model architecture, dataset size, and performance gains over training from scratch.

Through the studies performed, it is observed that the success of DTL strategies highly relies on the task to be performed. In medical image segmentation, it is mostly favored over the use of deep-based segmentation, since the segmentation demands accurate spatial feature learning and precision in pixel-based adaptation. The enhanced performance of Dice scores and boundary accuracy through fine-tuning of pre-trained encoders, including VGG-16, ResNet, and U-Net, have been observed through numerous studies in the area, particularly in segmentation problems of MRI and CT images [34–37, 41, 47, 52].

In contrast, medical image classification tasks often benefit more from feature extraction, particularly when the available labeled data are limited. Several classification studies have shown that freezing convolutional layers pre-trained on ImageNet and training only the classification layers reduces overfitting while maintaining high diagnostic accuracy [72, 76, 79, 82, 91]. In detection tasks, which are typically used in a hybrid approach, a mix of partial fine-tuning and feature extraction is generally adopted to balance generalization and learning, particularly when small or heterogeneous data is present [74, 75, 103]. These findings show that the best use of fine-tuning is in segmentation, whereas in classification feature extraction can be adequate and effective, and hybrid schemes are becoming common in detection-based applications.

Besides the use of transfer strategies, it is also possible to find consistent trends in performance across various pre-trained backbone architectures. VGG-based models have become popular because of their simplicity and usefulness in early medical imaging tasks, but their high number of parameters can easily limit scalability. The ResNet architectures are more stable and consistent in their performance on segmentation, classification, and detection tasks due to residual connections, which address the problem of vanishing gradients, especially in deep networks. DenseNet models have significant benefits on small and medium-sized datasets because of the effective feature sharing and enhanced gradient flow. Older architectures like EfficientNet have better accuracy-efficiency trade-offs, and more recently, Vision Transformer (ViT) models are becoming a promising direction, particularly with large-scale data and multi-modal medical imaging tasks, with better global context modeling in both MRI, CT, and histopathology images [34, 41, 52, 76, 82, 91, 100, 102].

Across the reviewed studies, DTL consistently outperforms training from scratch, particularly in scenarios involving limited annotated data. Quantitative comparisons reported in the literature indicate that DTL-based segmentation models achieve improvements of approximately 5–15% in the Dice similarity coefficient compared to models trained from scratch, especially in MRI and CT datasets. Similarly, classification tasks show average accuracy gains of 3–12% when pre-trained models are fine-tuned or used as feature extractors. In detection-based applications, reported improvements typically range from 4–10% in mean average precision (mAP). These performance gains highlight the effectiveness of knowledge transfer in accelerating convergence and improving generalization, thereby reducing reliance on large-scale labeled medical imaging datasets [34, 41, 47, 72, 76, 82, 91].

The effectiveness of DTL is also strongly influenced by dataset size. The reviewed studies indicate that DTL provides the most substantial performance gains on small-to-medium-sized datasets, where the number of annotated samples is typically fewer than several thousand. In such cases, TL mitigates overfitting and accelerates convergence by leveraging pre-learned representations. As dataset size increases, the relative performance gap between DTL-based models and those trained from scratch gradually narrows, particularly for classification tasks. However, no clear dataset-size threshold was identified below which DTL becomes ineffective; instead, its benefit diminishes progressively as the availability of labeled data increases. These observations suggest that DTL remains advantageous even for very limited datasets, although careful strategy selection (*e.g.*, feature extraction versus fine-tuning) becomes increasingly important in extremely small-sample scenarios [34, 41, 52, 72, 76, 82, 91]. There is no clear dataset-size threshold presented uniformly across the studies; however, based on the accuracy levels achieved by existing methods, it is believed that the benefits of DTL are most significant for datasets with small sample sizes (usually fewer than a few thousand images). Below this threshold for image size, DTL still offers benefits, but the decision on transfer strategy becomes more critical to prevent overfitting and negative transfer.

Across the reviewed literature, the majority of studies (approximately 60-80%) used data augmentation methods to address data scarcity and improve model generalization. A substantial proportion of the surveyed works incorporate data augmentation, particularly in segmentation and classification tasks involving limited datasets. The most commonly adopted techniques include geometric transformations such as rotation, flipping, scaling, and cropping, followed by intensity-based augmentations such as contrast adjustment, noise injection, and histogram equalization. More advanced augmentation strategies, including elastic deformation and generative adversarial approaches, are also reported in a smaller subset of studies. These findings indicate that data augmentation plays a complementary role alongside DTL, further enhancing model generalization when annotated medical images are scarce [35, 36, 47, 52, 72, 76, 82, 91].

Beyond conventional convolutional neural networks, recent studies increasingly explore advanced architectures such as Vision Transformers (ViTs), EfficientNet, DenseNet, and attention-enhanced models within DTL frameworks for medical imaging. Vision Transformers, in particular, have demonstrated strong potential for TL by effectively modeling long-range dependencies and global contextual information, especially in large-scale, multi-modal datasets, as reported in recent work [100, 102]. EfficientNet architectures offer improved trade-offs between accuracy and computational efficiency through compound scaling, making them suitable for resource-constrained medical imaging applications. DenseNet models facilitate effective feature reuse and improved gradient propagation, which is advantageous in small and medium-sized datasets. Additionally, attention mechanisms integrated into CNN backbones enhance feature representation by emphasizing clinically relevant regions, thereby improving segmentation and classification performance across various imaging modalities.

#### 4.4.1 Emerging Deep Transfer Learning Architectures and Recent Trends

Although early DTL work in medical imaging heavily depended on standard CNN frameworks (including VGG, ResNet, and Inception), more recent work has turned to more complicated and effective architectures. TL models based on dense nets have shown improved performance on small and medium medical datasets because dense features are recycled and gradients are propagated beneficially, especially for MRI and X-ray image classification [76, 82, 91]. The architectures of EfficientNet have been considered to offer the best accuracy-efficiency trade-offs with compound scaling, and thus are suitable for computationally constrained clinical tasks [100].

More recently, Vision Transformer (ViT) models have emerged as a promising transfer-learning paradigm in medical imaging, offering improved global context modeling compared to convolutional architectures. Pre-trained ViT models fine-tuned on medical datasets have shown competitive or superior performance in tasks such as brain tumor classification, histopathology image analysis, and multi-modal imaging, particularly when sufficient data or hybrid CNN–Transformer designs are employed [102].

Additionally, attention mechanisms integrated within CNN and hybrid architectures have demonstrated notable performance gains by enabling adaptive focus on clinically relevant regions, thereby improving interpretability and robustness in segmentation and classification tasks. These emerging trends indicate a gradual shift from purely convolutional TL frameworks toward hybrid and transformer-based architectures in modern medical image analysis.

#### 4.4.2 Key Empirical Findings

Based on the comprehensive analysis of the reviewed studies, several consistent empirical trends can be identified. Fine-tuning emerges as the most effective DTL strategy for medical image segmentation tasks, as pixel-level prediction requires strong task-specific feature adaptation. Feature extraction is particularly effective for medical image classification, especially when annotated datasets are limited, as it reduces overfitting while maintaining high diagnostic accuracy. For detection-based applications, hybrid strategies that combine partial fine-tuning with feature extraction offer a balanced trade-off between generalization and task specificity. Across architectures, ResNet and DenseNet backbones demonstrate stable and consistent performance across modalities, while EfficientNet offers favorable accuracy–efficiency trade-offs. Recent studies also indicate that Vision Transformer-based models are increasingly competitive in large-scale and multi-modal settings. Overall, DTL consistently outperforms training from scratch, particularly in small- and medium-sized medical imaging datasets, reinforcing its practical relevance in clinical applications.

The comparative trends discussed in this section are supported by the quantitative and qualitative summaries presented in Tables **1**–**3**, which consolidate performance metrics, model choices, and dataset characteristics across segmentation, classification, and detection tasks.

### 4.5 Reasons for Performance Variations in Deep Transfer Learning Approaches

The performance differences observed among DTL techniques across medical imaging tasks can be attributed to several architectural and learning-related factors. Fine-tuning strategies often yield superior performance in segmentation tasks by allowing deeper network layers to adapt to domain-specific spatial patterns and fine-grained anatomical structures, which are critical for pixel-level prediction. In contrast, feature extraction approaches typically perform well in classification scenarios with limited annotated data, as freezing early convolutional layers helps preserve robust low-level representations while reducing overfitting.

Network architecture also plays a crucial role in determining performance. Residual connections in ResNet-based models facilitate stable gradient propagation and enable effective training of deeper networks, thereby improving generalization across diverse imaging modalities. DenseNet architectures further enhance performance by promoting feature reuse and strengthening gradient flow, particularly in small- to medium-sized medical datasets. More recent models, such as EfficientNet, achieve high accuracy by balancing network depth, width, and resolution, offering improved efficiency without high computational cost.

Additionally, task complexity, dataset size, and domain similarity between source and target datasets significantly influence TL effectiveness. When the source domain closely resembles the target medical imaging domain, knowledge transfer becomes more effective, resulting in faster convergence and higher accuracy. Conversely, large domain shifts or highly heterogeneous datasets may reduce performance due to negative transfer, necessitating careful selection of transfer strategies and fine-tuning depth. These factors collectively explain why certain DTL approaches consistently outperform others on specific medical imaging tasks.

Based on the comprehensive review of the studies under consideration, several significant empirical observations can be outlined. Segmentation is always best performed using the fine-tuning approach, which allows it to be tailored to the domain's spatial characteristics. Conversely, feature extraction has been extensively applied to classification problems with limited data, as it can minimize overfitting without sacrificing performance. Detection tasks are typically performed using hybrid methods to balance generalization and task-specific learning.

ResNet and DenseNet models exhibit more predictable, stable performance across tasks than the previously used VGG-based models. EfficientNet offers higher performance and greater computational efficiency, whereas transformer-based models have yet to prove their potential on large-scale, multimodal datasets. Also, DTL consistently outperforms training from scratch, especially on small and medium-sized datasets.

## 5 CHALLENGES AND OPPORTUNITIES IN DEEP TRANSFER LEARNING TECHNIQUES IN MEDICAL IMAGE PROCESSING AND ANALYSIS

Medical image processing with DTL is a rapidly developing field that solves complex healthcare problems, including disease classification, segmentation, and sickness detection, using pre-trained DL models. Although there are still many obstacles to overcome, it has demonstrated incredible promise in improving the level of accuracy and effectiveness of medical image processing. However, other special chances might be used to advance the field. By applying the ability to apply information from previously trained models to specific medical tasks, DTL has emerged as a groundbreaking tool in medical imaging. Despite its advantages, certain problems need to be addressed. It has many exciting potential applications in medical imaging and is revolutionizing the diagnosis and treatment of several illnesses. DTL increases diagnostic precision and effectiveness by using pre-trained models trained on large datasets to improve medical image analysis accuracy and efficiency. DTL is applicable to various other challenging applications, such as video classification, social network analysis, and logical inference, and is growing in unsupervised TL [6]. Knowledge transfer between imaging modalities, such as CT and MRI, is known as cross-modality integration and can yield comprehensive diagnostic models that leverage each modality's strengths [128]. The opportunities and difficulties encountered by DTL techniques in medical image processing are summarized below.

### 5.1 Opportunities of DTL in Medical Image Processing and Analysis

#### 5.1.1 Synthesizing Data and Augmenting Data

The lack of labeled data may be addressed by using synthetic data generation methods, such as Generative Adversarial Networks (GANs), to produce artificial medical images that closely resemble real-world data. In a TL scenario, these artificial images can improve model performance and resilience, and data augmentation techniques, including cropping, scaling, and rotation, can help expand small datasets and improve their suitability for DTL.

#### 5.1.2 Domain Adaptation

To address domain shift, researchers are investigating domain adaptation strategies. In medical image processing, techniques such as adversarial training, unsupervised domain adaptation, and fine-tuning using domain-specific pre-training can increase the effectiveness of TL. Additionally, Negative transfer can be reduced, resulting in more successful TL, by emphasizing the acquisition of generalizable characteristics and avoiding overfitting to the source domain.

#### 5.1.3 Federated Learning

Recent studies further highlight the feasibility of privacy-preserving decentralized learning frameworks. For example, federated learning-based approaches enable collaborative model training across institutions without sharing raw data, thereby maintaining patient confidentiality while improving model robustness. One potential remedy for data privacy issues in medical imaging is federated learning, in which models are trained cooperatively across institutions without exchanging raw data. The risk of data breaches can be reduced by medical institutions collaborating to train a shared model while maintaining on-site security for sensitive patient data. To enhance a common model and collaborate across many medical institutions and geographical regions while adhering to privacy rules and data-sharing agreements, TL makes this easier. Using DTL in federated learning frameworks enables models to learn from decentralized data sources while protecting patient privacy. It can be used in various industries to promote data integration while adhering to data privacy and security standards. Instead of merely concentrating on improving model performance, it seeks to improve data integration techniques [129].

#### 5.1.4 Few-shot Learning

The goal of few-shot learning techniques is to learn from a limited number of labeled instances. In medical imaging, where obtaining sizable labeled datasets can be costly or challenging, this is extremely helpful. Models may transfer information from a task in the source to a task in the target and generalize from sparse data through few-shot learning.

#### 5.1.5 Integration with Emerging Healthcare Technologies

The integration of DTL with emerging healthcare technologies, such as cloud computing and the Internet of Things (IoT), offers significant opportunities for scalable, real-time medical image analysis. Cloud-based platforms can provide the computational resources required for training and deploying DTL models, enabling resource-constrained healthcare facilities to access advanced diagnostic tools. Similarly, IoT-enabled medical devices and imaging systems can facilitate continuous data acquisition and remote monitoring, allowing TL models to adapt dynamically to evolving clinical data. Such integrations have the potential to support real-time decision-making, telemedicine, and large-scale collaborative healthcare applications.

#### 5.1.6 Real-Time Diagnostic Applications

DTL also offers promising potential for real-time medical image analysis to support immediate clinical decision-making. By leveraging pre-trained models and efficient fine-tuning strategies, DTL-based systems can enable rapid inference for tasks such as disease screening, lesion detection, and emergency diagnosis. When combined with optimized architectures and edge or cloud-based deployment, real-time DTL applications can assist clinicians by providing fast and reliable diagnostic insights, particularly in time-sensitive scenarios such as stroke assessment, trauma evaluation, and critical care imaging.

Emerging trends in AI are expected to further enhance the clinical impact of DTL in medical imaging. In particular, interpretable and explainable AI models are gaining importance for improving clinician trust, supporting prognostic assessment, and enabling multimodal data integration. Recent advances in large-scale models, including transformer-based architectures and large language models, also demonstrate promising potential for handling complex clinical workflows, multimodal reasoning, and decision support in medical imaging. Integrating these emerging AI paradigms with DTL frameworks may significantly improve diagnostic accuracy, interpretability, and personalized clinical decision-making.

### 5.2 Challenges of DTL in Medical Image Processing and Analysis

DTL offers promising medical image processing that improves diagnostic accuracy and enables real-time analysis. Despite the benefits of DTL, several challenges arise when training models, which are as follows:

#### 5.2.1 Domain Shift

According to a previous study [130], the domain shift problem is a significant challenge in medical image analysis. A “domain shift” is a difference in the overall distribution of data between the training dataset (source domain) and the test dataset (target domain). Model performance may suffer when this mismatch occurs during use on data collected recently and different from the training data. Domain shift is especially common in medical imaging because different medical institutions have varying patient demographics, scanning methods, and imaging equipment. These factors affect differences in visual quality and intensity, which makes it more difficult for models to generalize.

Besides performance degradation, domain shift also creates issues with data compatibility, as variations in image resolution, acquisition protocols, scanner vendor, and annotation standards make it difficult to effectively transfer knowledge. These kinds of incompatibilities can restrict the generalization of models across institutions and modalities and require careful preprocessing, normalization, or domain adaptation to ensure reliable Implementation in the clinical environment.

#### 5.2.2 Data Availability

One issue with applying DTL to medical image analysis is the lack of data. The absence of large-scale annotated datasets can be an impediment to medical image analysis. Providing good training to DL models at the outset is problematic due to this scarcity. When using TL, particularly during fine-tuning, overfitting may occur if the model has more parameters than the number of available samples in the target dataset. This could lead to poor model generalization [131]. DL models are computationally demanding, particularly when scaling to huge datasets. This can be a challenge for many healthcare organizations that lack the appropriate hardware. Modest entities may encounter difficulties in implementing these technologies.

#### 5.2.3 Data Complexity

In addition to data scarcity, data complexity plays a crucial role in determining the performance of DTL models, particularly in high-dimensional medical imaging modalities such as brain MRI. Recent studies have evaluated the dependence of DL models on data complexity for the classification of neurological disorders, including brain tumors and Alzheimer’s disease. These analyses reveal that variations in anatomical structures, disease progression stages, and imaging characteristics significantly influence feature representation and model generalization. These findings indicate that TL effectiveness is governed not only by data volume but also by the intrinsic complexity of medical imaging data, underscoring the need for complexity-aware feature extraction and adaptive transfer strategies in neuroimaging applications.

#### 5.2.4 Negative Transfer

Negative transfer occurs when source-domain information negatively affects learning in the target domain, leading to poor performance, whereas positive transfer occurs when source-domain information enhances learning in the target domain. DTL models may not always be trainable and fine-tunable due to their high resource needs and computational complexity. Lack of accessibility may render the medical personnel less confident [132]. The work at higher levels is specialized to the original work, and this influences performance when transferred to new tasks. Such a loss of generality prevents the model from adapting. Moreover, cross-adapted neurons in adjacent layers could act as a barrier to the effective transfer of features. Dependency between layers can be either feature-specific or difficult to optimize, and the less dissimilar the tasks are, the less transferable are their dependencies [133].

#### 5.2.5 Overfitting

Pre-trained models are often easily optimized using the target medical dataset, but this can lead to overfitting, especially when the target dataset is small or not diverse enough. Overfitting reduces the model's ability to generalize to new, unobserved medical data. The DenseNet model is effective for training and provides flexibility for training various tasks with minimal data, which is important in TL. It has fewer parameters to reach high accuracy and is hence parameter-efficient. Tense connections decrease overfitting, especially when using smaller datasets. They have shown significant improvements over state-of-the-art models by effectively leveraging pre-trained weights, making them a strong competitor to TL. The above prospects demonstrate how the model can enhance the use of DTL in a range of fields [134].

A number of studies have reported that overfitting is especially severe when DTL models are fine-tuned on small or homogeneous medical datasets, resulting in worse performance on unseen data. The literature has widely embraced architectural decisions to reduce this problem, including ensuring dense connectivity between layers, using regularization strategies, selectively freezing layers, and augmenting data to enhance model robustness.

#### 5.2.6 Data Security and Privacy

Due to the extreme sensitivity of medical data, confidentiality regulations must be followed. A major challenge is the safe exchange of data for DL model training, particularly when patient data is involved. DTL has demonstrated potential to improve model performance across multiple domains. These strategies improve the robustness of models trained on limited datasets by combining TL with enhanced data augmentation, thereby improving model performance and reducing overfitting to unknown data [135]. However, barriers must be overcome by carrying out further studies on these possibilities, and interdisciplinary cooperation is essential to achieving its revolutionary effects. In federated TL settings, additional technical challenges arise from non-independent and non-identically distributed (Non-IID) data across participating medical centers. Variations in patient demographics, imaging protocols, and scanner characteristics can negatively affect model convergence and performance stability. Addressing these issues requires strategies such as personalized federated learning, adaptive aggregation, and domain-aware fine-tuning to ensure effective knowledge transfer while preserving data privacy.

#### 5.2.7 Privacy-Preserved and Decentralized Learning

Recent studies on privacy-preserving and decentralized learning have demonstrated the feasibility of training DL models without centralized data sharing in medical imaging applications. In particular, Al-Saleh *et al*. (2025) [136] proposed a federated learning-based privacy-preserving framework for brain tumor detection from CT scans, where models are collaboratively trained across multiple institutions while keeping patient data localized. This approach effectively addresses data privacy concerns and regulatory constraints by eliminating the need for raw data exchange, while still achieving competitive detection performance. Such federated and decentralized DTL paradigms offer a promising pathway for large-scale, multi-institution medical imaging studies, enabling robust model generalization without compromising patient confidentiality.

## CONCLUSION

TL has emerged as one of the most prominent DL techniques and has evolved into DTL to address challenges such as achieving high diagnostic accuracy, limited labeled data availability, and high computational complexity in medical image analysis. By leveraging pre-trained models trained on large-scale datasets, DTL enables effective knowledge transfer to specific clinical domains, thereby enhancing performance in medical image classification, segmentation, and disease detection. This review demonstrates the effectiveness of DTL across a wide range of medical imaging modalities, including X-ray, ultrasound, CT, and MRI. The primary focus of this review is on network-based DTL approaches, including feature freezing, fine-tuning, federated learning, and progressive learning strategies. Federated learning is discussed as a privacy-aware network-based DTL paradigm that addresses data security concerns in collaborative medical imaging applications. Furthermore, more than 100 research studies published between 2017 and 2025 were systematically reviewed and categorized by imaging task and learning strategy. The analysis indicates that fine-tuning remains the most widely adopted and effective DTL strategy. Despite its promising performance, several challenges remain, including ensuring model reliability and interpretability, addressing data privacy concerns, and improving model adaptability across diverse clinical scenarios. Future research should focus on developing interpretable, clinically reliable DTL models to improve transparency in medical decision-making. The integration of multimodal data, including imaging, clinical, and genomic information, is expected to further enhance diagnostic and prognostic capabilities. In addition, privacy-preserving frameworks such as federated and decentralized learning will play a critical role in large-scale collaborative medical applications. Emerging directions such as few-shot learning, self-supervised learning, and real-time deployment of DTL models also offer significant potential for improving scalability and clinical applicability.

## Figures and Tables

**Fig. (1):**
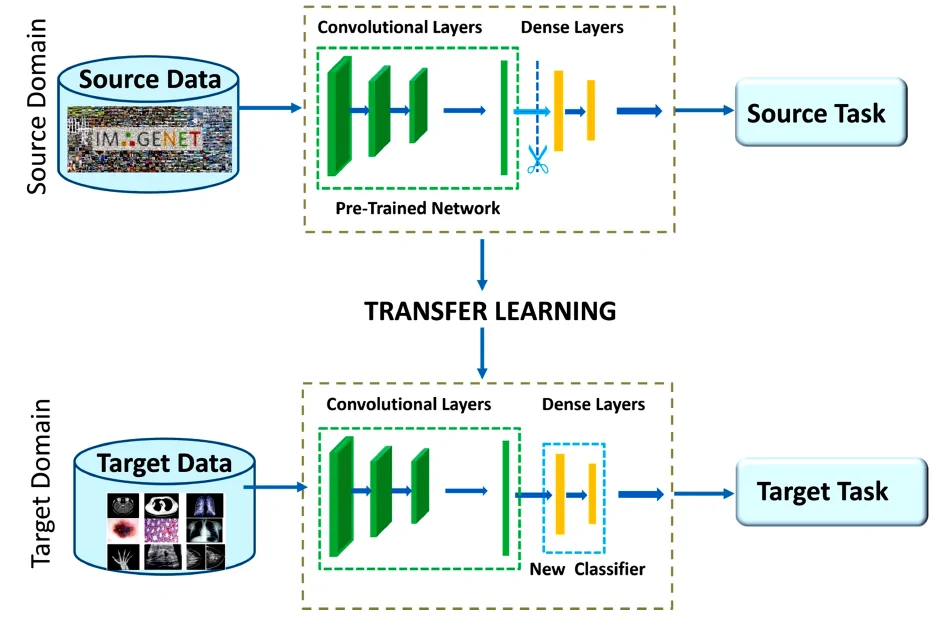
Outline of transfer learning.

**Fig. (2):**
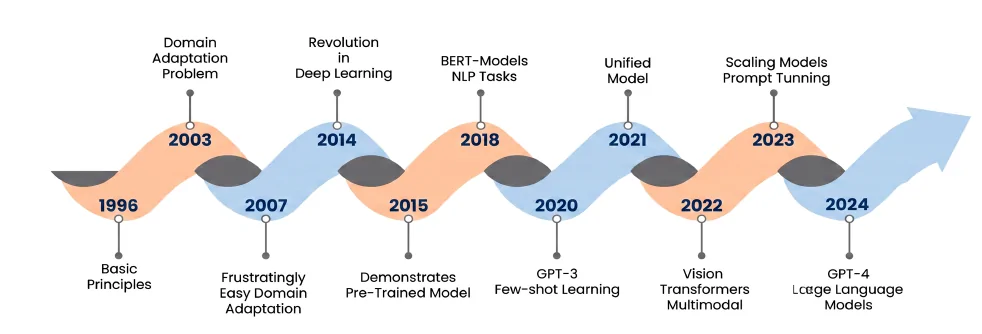
Historical evolution of transfer learning.

**Fig. (3):**
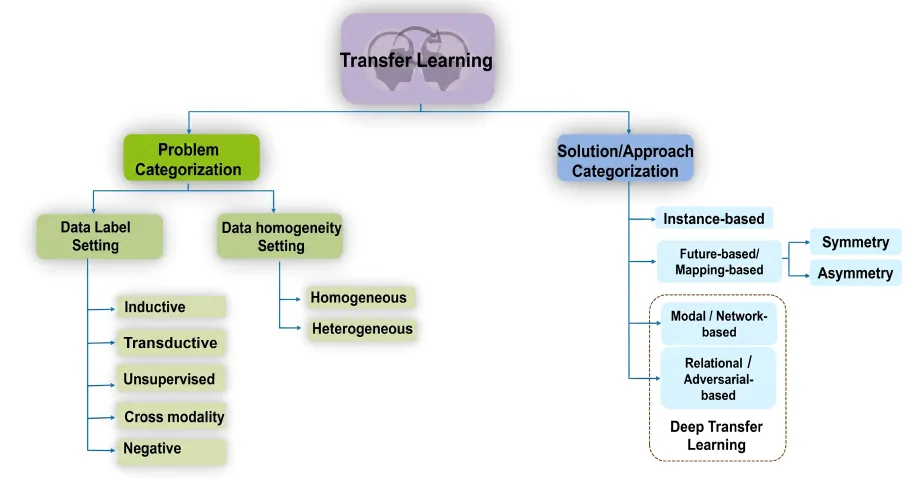
Conceptual taxonomy representation of transfer learning, applicable to deep transfer learning.

**Fig. (4):**
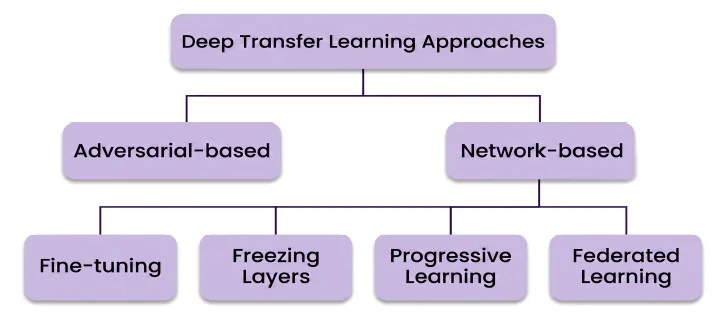
Implementation-level categorization of DTL approaches.

**Fig. (5):**
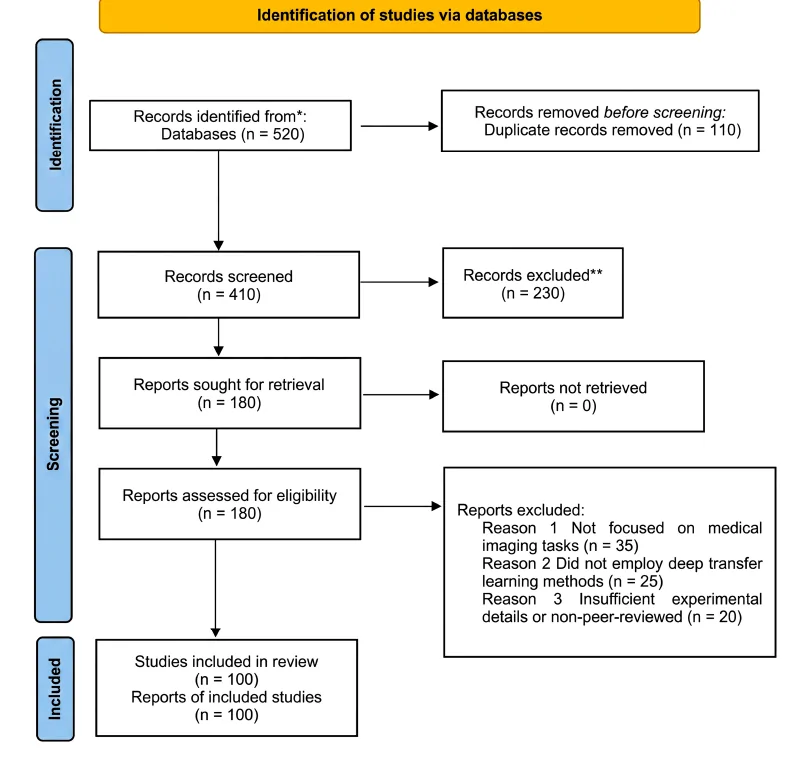
PRISMA-style flow diagram of the literature search and study selection process. * Records were identified from multiple databases. ** Records were excluded based on title and abstract screening.

**Fig. (6):**
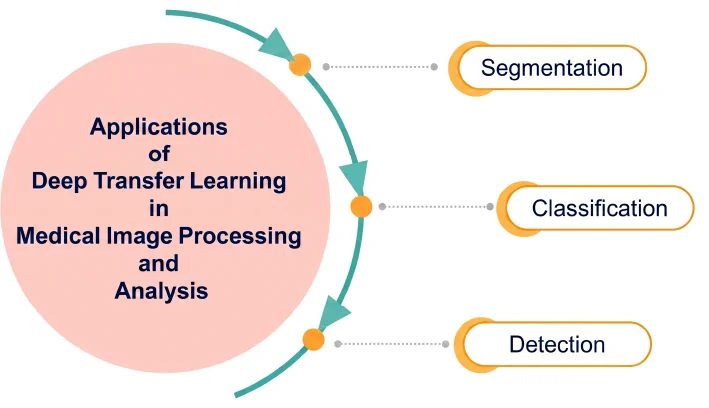
Applications of DTL in medical image processing and analysis.

**Table 1:** Summary of several deep transfer learning segmentation techniques in medical image processing.

**Reference**	**Imaging Modality used**	**Focused Regions**	**Pre-trained model adopted**	**Tasks Undertaken**	**Significance**	**Limitations**
Xu *et al*. (2017) [27]	MRI (MICCAI -NeoBrainS12, MRBrainS13)	Brain (Tissues)	VGG16	Segmentation of tissues by fine-tuning with the use of ImageNet.	The segmentation running time was greatly decreased.	Limitations are not stated clearly.
Xu *et al*. (2018) [28]	MRI (MICCAI WMH)	Brain (White Matter Intensities)	VGG16	Lesion segmentation by fine-tuning along with ImageNet.	Though no training data was available for the GE 1.5T, the suggested approach still functions well with that data.	Some lesions are still missed by the proposed method.
Oliveira and Dos, (2018) [29]	X-ray (JSRT)	Chest (Lungs, Heart & Clavicle)	U-Net	Segmentation of multi-organs *via* fine-tuning using the JSRT dataset weights.	The proposed method outperformed training from scratch and fine-tuning while working with TL.	Additional training time with more GPU memory. Metrics indicate that the model did not successfully reach convergence.
Hamed Mozaffari and Lee (2019) [30]	US (uOttawa, Seeing Speech)	Tongue	VGG-net	Contour extraction *via* fine-tuning along with ImageNet weights	This qualitative study demonstrates that domain adaptation can enhance segmentation outcomes for frames with notable noise and artifacts by better generalizing the network over various cross-domain samples.	A typical ultrasound tongue dataset is no larger than 200 K frames, in contrast to other research fields with large datasets.
Amin *et al*. (2019) [31]	MRI/CT ((BRATS) 2013- 2017, ISLES (2018))	Brain	AlexNet, GoogleNet	Segmentation and classification are done by fine-tuning and feature extraction using ImageNet.	The suggested model accurately identifies tumors in any imaging view, including axial, coronal, and sagittal.	Only entire tumor regions are segmented or classified; sub-regions are not.
Chen *et al*. (2019) [32]	MRI and CT	Brain	ResNet, Med3D	Segmentation of organs through feature extraction using Med3D Datasets.	Med3D improves training speed for 3D medical tasks by two times that of Kinetics pre-training and ten times that of training from scratch.	Quality variations occur when data is collected from many sites.
Soudani & Barhoumi, (2019) [33]	Dermatoscopy (ISIC2017)	Skin	VGG16 and ResNet50	Segmentation of lesions done through feature extraction along with ImageNet	Introducing a segmentation recommender with prediction capabilities based on crowdsourcing and transfer learning.	Precision and unpredictability in manual inspection are restricted.
Puybareau *et al*. (2019) [34]	GE-MRI (MICCAI 2018)	Heart (Left Atrial)	VGG-16	Segmentation of the left atrial cavity *via* fine-tuning with the help of ImageNet.	The suggested technique examines a whole volume in less than three seconds, greatly increasing the speed of left atrial cavity segmentation from 3D GE-MRIs.	The proposed technique only came in third place with a Dice score of 0.923.
J. Lee and Nishikawa (2020) [35]	Mammogram (BI-RADS, DDSM), MRI (ISBI), X-ray	Breast, Brain (White Matter), Prostate,	CNN, VGG16, ResNet18	Segmentation of lesions and classification of tumors *via* fine-tuning with ImageNet	The experimenter presented that a DL model trained using mammograms can be adapted for various medical images effectively, even though there are major variations in different imaging modalities and regions, like 2D versus 3D, MRI versus X-ray, breast versus prostate, and brain.	The XTL CNN's performance on MR lesion segmentation degraded due to fewer samples available for intermediate training.
Nazi and Abir (2020) [36]	Dermatoscopy (PH 2, ISIC 2018)	Skin	VGG16, VGG19, InceptionResNetV2, ResNet50, Xception Inception V3 and DenseNet201	Segmentation of lesions and detection of melanoma was performed using feature extraction along with ImageNet and fine-tuning.	Since U-Net was trained using additional augmented training images, it performed well even with spatial dropout.	The lack of labeled Dermatoscopy images reduces the efficiency of training.
Vu *et al*. (2020) [37]	CT	Chest (Multi-Organ)	VGG 16	Segmentation of organs using ImageNet.	A 2D U-Net model combined with non-domain TL can accurately segment organs-at-risk (OAR).	Ground-truth labeling varies due to numerous radiation oncologists.
Lizancos Vidal *et al*. (2021) [38]	X-ray (General, CHUAC)	Chest (Lungs)	U-net	Segmentation of lungs through feature extraction with MRI of cerebral glioma.	A fully automated deep image-based approach has been successfully developed to process reliable images on portable devices.	Image quality was impacted by a slight loss in smoothness and by inaccuracies in rescaling.
Cheng and Lam (2021) [39]	US (XPIE, Real-time)	Chest (Lungs)	VGG-16, U-net	Segmentation by fine-tuning with VGG-16 and freezing the X-Unet are pre-trained using ImageNet and XPIE, respectively.	Although the DICE coefficient was lower, the X-Unet model produced correct predictions when pretrained with grayscale images.	No shortcomings are mentioned here.
Karimi *et al*. (2021) [40]	CT & MRI	Multi Organs	FCN, HRNet, UNet++, Tiramisu, Autofocus	Lesion and Tumor segmentation *via* fine-tuning using their own pre-trained datasets.	Feature reuse in TL may be more important at deeper layers and the network's decoder part, depending on the source and target domains.	Networks learned from scratch and networks trained by TL were compared in this work using only a scalar similarity metric.
J. Liu *et al*. (2021) [41]	CT (LIDC-IDRI, MosMedData, Coronacases)	Lungs	ResNet50	Segmentation of infection by feature extraction along with ImageNet	The suggested approach surpasses state-of-the-art segmentation models and achieves promising performance in lung infection segmentation.	Limitations are not stated clearly.
Hsiao *et al*. (2021) [42]	CT (KiTS19, LiTS17, and private dataset (NTU))	Kidney	ResNet 50, VGG 16	Segmentation of Kidney through feature extraction by LiTS17 and NTU dataset weights.	The lightweight ResNet-50 encoder using the suggested approach with TL obtained a Dice score of 0.9689.	No limitations are mentioned.
Ankenbrand *et al*. (2021) [43]	Cardiac Cine-MRI (Kaggle)	Heart	ResNet34, ResNet50, and VGG16	Segmentation of cardiac cine images by weight initializing of ImageNet.	For myocardium and left ventricle segmentation, the DICE scores were considerably raised by TL.	A common benchmark dataset is required.
Zhu *et al*. (2022) [44]	CT (Private, KiTS19)	Kidney	U-Net	Segmentation and classification of tumors by feature extraction using an auxiliary dataset.	The study showed that the effectiveness of TL in medical imaging utilizing FS-Net for accurately segmenting kidney tumors in 3D CT images, attaining outstanding performance compared to traditional methods.	Results are not as generalizable with a small sample size. The different tumor subtypes have different performances. Analysis requires additional patient images.
Araújo *et al*. (2022) [45]	Dermatoscopy (PH2, ISIC 2018, and DermIS)	Skin	U-Net and LinkNet	Segmentation of melanoma is done through fine-tuning using ImageNet.	The study showed accurate segmentation with small datasets and unbalanced classes.	The authors did not study the impact of pre-processing on computing cost reduction.
Maiello *et al*. (2022) [46]	CT	Chest (Lungs)	U-Net	Segmentation of lungs *via* fine-tuning along with U-Net pre-trained weights.	This suggested model provides appropriate estimations of lung volumes, aeration compartment sizes, and recruitability in animals and patients with various lung diseases.	The decreased similarity between the corresponding scans Only experimental models of reversible atelectasis were used to collect animal data.
Ambesange *et al*. (2023) [47]	X-ray	Lungs	U-Net	Segmenting lungs by using the pre-trained U-Net weights of MRI through fine-tuning	The suggested approach is a data-driven technique which builds the delivery of high-quality, unbiased data, among others, and is likely to continuously expedite the process of model creation and provide a higher level of performance.	The main work was performed in data organization, data processing, and the successful application of the TL in comparison to a model-driven approach; the most significant time was spent on building customer-oriented models over the dataset.
Riaz *et al*. (2023) [48]	CT (MSD, TCIA)	Lungs	MobileNetV2	Segmentation *via* feature extraction and fine-tuning by using ImageNet.	The proposed method performs better than current networks in terms of precision, recall, and dice score.	Only the challenge's validation set was used to evaluate our suggested segmentation technique.
Boyd *et al*. (2023) [49]	MRI (CBTN, BCH, BraTS 2021)	Brain	nnUNet	Segmentation *via* fine-tuning with adult model weights.	Highly accurate segmentation with a Dice score of 0. 877.	Adult-centric models perform badly in pediatric settings.
Sharma *et al*. (2023) [50]	MRI	Gastrointestinal Tract	Inception V3, SeResNet50, VGG19, DenseNet121, InceptionResNetV2, and EfficientNet B0.	Segmentation of the GI tract is done by fine-tuning with ImageNet.	The U-Net model improves the segmentation of gastrointestinal organs for cancer treatment.	Limited research has been conducted on the segmentation of GI tract organs.
Ali and Agrawal (2024) [51]	MRI (Kaggle)	Brain	VGG-16, VGG-19, ResNet50V2, ResNet101V2, and InceptionV3	Segmentation of tumors through feature extraction with ImageNet weights.	Research tackles the problem of segmenting different types of brain tumors.	ResNet101 does not utilize the VGG19 stack for feature extraction.
Ren *et al*. (2024) [52]	mp-MRI (BraTS 2023)	Brain	U-Net	Segmentation of tumor *via* fine-tuning with the glioma dataset in Challenge 1.	Combining Dice, Focal, and Edge loss improved evaluation scores.	'Dust' is present in high quantities in ground truth segmentations. Data needs to be both more and better in quality.
Srivastava *et al*. (2024) [53]	MRI (BraTS 2019)	Brain	VGG16, ResNet50, InceptionV3, MobileNetV2 and DenseNet121	Segmentation and classification through feature extraction along with ImageNet.	The Custom CNN model achieves high accuracies using TL.	There are no limitations mentioned.
Oostrom *et al*. (2024) [54]	Light- Sheet Microscopy	Brain (Axon)	TrailMap, 3D U-Net	Segmentation of Axons by performing fine-tuning with new data.	The proposed work optimizing TL improves the TrailMap model for axon segmentation.	Optimized models might not be able to generalize to other brain regions.
Basha *et al*. (2024) [55]	MRI (TCIA)	Brain	Inception V3, VGG-16, and ResNet-50	Segmentation of tumors through feature extraction using ImageNet	The proposed work achieves 99% sensitivity and accuracy in tumor detection.	There were no shortcomings mentioned in this paper.
Clark *et al*. (2024) [56]	CT	Head and Neck	Public Model	Segmentation through fine-tuning using a public dataset	TL from public models enhances performance on institutional data sets.	The model's performance was not qualitatively assessed.

**Table 2:** Summary of various deep transfer learning classification approaches for the processing of medical images.

**Reference**	**Imaging Modality used**	**Focused Regions**	**Pre-trained model adopted**	**Tasks Undertaken**	**Significance**	**Limitations**
Samala *et al*. (2017) [58]	Mammogram (SFM, DM)	Breast (Mass)	VGG, ZFnet, GoogLeNet and Microsoft ResNet	Classification of lesions through fine-tuning using ImageNet	Multi-task supervised learning outperformed single-task learning regarding generalization to unknown cases when transfer learning for DCNNs was utilized.	The DCNN structure's layers, nodes, and hyperparameters were not compared, nor were the results of the DCNN's performance compared to those of traditional machine learning methods.
Meng *et al*. (2017) [59]	US (ILSVRC)	Liver	VGG	Classification of fibrosis is done by fine-tuning and feature extraction using ILSVRC.	Effective liver status classification from US images is achieved using TL using VGGNet.	Many small elements affect the quality of the image.
Li *et al*. (2018) [60]	fMRI (ABIDE)	Brain	Stacked Sparse Autoencoder (SSAE)	Classification of brain functional connectomes *via* fine-tuning using pre-training ABIDE datasets	The outcomes provide evidence that suggested models can be used instead of traditional models to improve relatively rare disease diagnosis or prediction.	Learned knowledge may not apply to other features. Determining how many healthy FC patterns are needed in transfer learning to attain optimal performance is challenging because categorization problems vary widely in complexity.
Byra *et al*. 2019 [61]	US (UDIAT, OASBUD)	Breast	CNN, VGG19,	Classification of Breast Mass by feature extraction and fine-tuning with ImageNet weights.	A greater AUC was attained by a classifier that performed better.	Limitations are not made explicit.
Ahmed *et al*. (2019) [62]	US (Baheya Foundation, Egypt)	Breast	VGG16	Lesion Classification by fine-tuning using ImageNet	Even though our dataset is very small, the results claim that the optimized model using medical data for pre-training has increased the classification accuracy.	No limitations are explicitly mentioned.
Samala *et al*. (2019) [63]	Mammogram (SFM, DM), DBT	Breast	AlexNet	Classification of breast cancer *via* fine-tuning using ImageNet weights.	It may be too optimistic to provide the best result from thorough searches using a “test” collection.	Because of the limited amount of data sets available and the associated computational expenses, authors did not try to “optimize” the hyperparameters.
Swati *et al*. (2019a) [64]	CE-MRI (Public)	Brain	AlexNet, VGG16, and VGG19	Classification of tumors through fine-tuning with ImageNet weights.	The classification model using transfer learning and fine-tuning outperforms classical approaches with an accuracy of 94. 82% under cross-validation.	Risk of overfitting because of small dataset size
Talo *et al*. (2019) [65]	MRI (Private)	Brain	ResNet34	Classification *via* fine-tuning along with ImageNet.	Complicated layer parameter computation costs, lengthy validation, and physical feature extraction are eliminated.	The study only makes use of a few images.
Deepak and Ameer (2019) [66]	MRI (figshare)	Brain	GoogleNet	Classification of tumors *via* feature extraction using ImageNet.	The suggested approach outperforms current approaches regarding average F-score, specificity, and sensitivity.	The transfer learning model fared poorly as a single classifier.
Yamada *et al*. (2019) [67]	DCE-CT	Liver	Alexnet, VGG-16, VGG-19, GoogLeNet, Inception-v3, ResNet-50, ResNet-101	Classification of liver cancer using ImageNet.	TL using pre-trained CNNs is effective for identifying liver cancer despite picture misregistration.	Some CNNs were not resilient enough to misregister skewly.
Loey, *et al*. (2020) [68]	X-ray	Chest (Lungs)	Alexnet, Googlenet, and Restnet18	Classification of COVID-19 *via* fine-tuning using ImageNet.	Googlenet has achieved 99.9% validation accuracy and 100% testing accuracy.	Insufficient datasets are accessible.
Mehrotra *et al*. (2020) [69]	MRI (TCIA)	Brain	AlexNet, GoogLeNet, SqueezeNet, ResNet50, and ResNet101	Classification of a tumor through transfer learning with ImageNet	The suggested work attains a remarkable 99.04% categorization accuracy.	Varying optimizers have drastically varying training times.
Rehman *et al*. (2020) [70]	MRI (Figshare)	Brain	AlexNet, GoogLeNet, and VGGNet	Classification of tumors *via* fine-tuning and freezing the CNN layers with ImageNet.	This work obtained 98.69% accuracy with fine-tuned VGG16.	Minimal research on transfer learning strategies for brain tumors.
Kaur and Gandhi (2020) [71]	MRI (Figshare, Private)	Brain	Alexnet, Resnet50, GoogLeNet, VGG-16, VGG-19, Resnet 101, Inceptionv3, and InceptionResNetV2	Classification through fine-tuning using ImageNet	The suggested work obtains 100%, 94%, and 95.92% classification accuracy for three separate datasets by using the pre-trained AlexNet.	High system requirements and a long computation time
Loey *et al*. (2020) [72]	Microscopy	Blood	AlexNet	Classification of blood cells *via* feature extraction and fine-tuning using ImageNet.	The second model outperformed the previous model and demonstrated the approach's usefulness, achieving an impressive 100% classification accuracy.	Not specified any limitations.
Rodrigues *et al*. (2020) [73]	Dermatoscopy (PH 2, ISBI-ISIC)	Skin	VGG16, VGG19, InceptionV3, ResNet50, Xception and MobileNet	Classification of lesions through feature extraction by ImageNet.	DenseNet201 and KNN obtained an accuracy of 93. 167% for the PH 2 dataset and 96. 805% for the ISBI-ISIC dataset.	Lesion similarities make classification difficult. High costs restrict Dermatoscopy device access.
Sun *et al*. (2020) [74]	WSI (TCGA)	Liver	ResNet 50	Classification of liver cancer *via* feature extraction using ImageNet.	TL and MIL enhance patch-level feature extraction.	Constrained to patch-level labels rather than global labels. Complex clinical symptoms hamper accurate classification.
Gu *et al*. (2020) [75]	Dermatoscopy, Normal Camera (MoleMap, HAM)	Skin	CNN	Classification of lesions through fine-tuning using ImageNet.	A two-step progressive transfer learning method that improves the model's performance by showing better generalization skills by fine-tuning several datasets.	Performance declines with different datasets because of bias and variation.
Ayana *et al*. (2021) [76]	US (OASBUD, UDIAT, BK Medical Panther 2002, BK Medical Hawk 2102)	Breast	VGG16, VGG19, AlexNet, and Inception V3	Lesion and Tumor classification through feature extraction and fine-tuning with ImageNet	The fine-tuned model performed the best. Transfer learning, which uses a model that has already been extensively trained on a huge dataset, produces better outcomes than cutting-edge techniques.	To attain better performance, there are still problems with the pre-processing techniques and dataset types for the target and training tasks.
Gour and Khanna (2021) [77]	Fundus (ODIR)	Eye	ResNet, VGG-16, Inception V3, MobileNet	Classification of Ophthalmological diseases *via* fine-tuning with ImageNet	Compared to CNN architectures like InceptionV3, ResNet, and MobileNet, the VGG16 network performs better.	After several training epochs, validation accuracy remains unchanged, despite Model-2 achieving high training accuracy with these architectures.
Aftab *et al*. (2021) [78]	Microscopy (Private)	Blood (Leukemia)	GoogleNet	Classification of Leukemia *via* feature extraction by using the given datasets (AML, ALL, CML, and CLL)	Deep learning with Spark improves disease diagnostic accuracy.	Few research studies on big data frameworks for disease prediction.
Aswathi *et al*. (2021) [79]	Fundus photography (Messidor)	Eye	VGG19, InceptionV3, ResNet50, MobileNet and NASNet	Classification of DR by feature extraction along with ImageNet.	High sensitivity and specificity were attained in a non-clinical setting by the suggested work.	The model's performance declines as the number of classes increases.
Houby (2021) [137]	Fundus photography (Kaggle)	Eye	VGG-16	Classification of Diabetic Retinopathy through feature extraction using ImageNet.	The method could identify anomalies in a binary classification test with a 75.5% accuracy rate.	The degree of classification accuracy varies greatly depending on the severity level.
Islam *et al*. (2022) [80]	X-ray (Mendeley Data)	Chest	VGG19, DenseNet121, InceptionV3, and Inception-ResNetV2	Classification of COVID-19 *via* feature extraction using ImageNet weights	Regarding detecting COVID-19 cases, the VGG19-RNN is considered the best model with 99.9% accuracy, 99.9% AUC, 99.8% recall, and 99.8% F1-score.	The experiment only correctly identifies chest X-rays from the posterior-anterior perspective,
Lee and Aqil (2022) [81]	Microscopy (Kaggle, HuBMAP, and Mendeley)	Kidney	ResNet101V2, ResNet50V2, VGG19, EfficientNetB7, and EfficientNetL2	Classification of glomeruli *via* fine-tuning with ImageNet and noisy student weights.	Utilizing transfer learning techniques and the ResNet101V2 model, the research improves renal glomerular tissue disease detection by achieving 97% accuracy in kidney glomeruli image classification.	Certain model combinations are not accurate. Predictions made by the EfficientNet-L2 model that are not effective.
Hasan *et al*. (2022) [82]	Dermatoscopy (ISIC-2016,2017, 2018)	Skin	CNN-based DSNet	Classification of lesions *via* feature extraction and fine-tuning with ImageNet weights.	The suggested work achieved AUC values of 0.96, 0.95, and 0.97 for the ISIC-2016, ISIC-2017, and ISIC-2018 datasets.	CNN training suffers from vanishing gradients and overfitting.
Khaled *et al*. (2022) [83]	X-ray (Kermany)	Chest	AlexNet, VGG-16, ResNet 50, DenseNet-121 and InceptionV3	Classification of pneumonia *via* fine-tuning along with ImageNet and through progressive transfer learning using the tuberculosis image dataset	Combining several pre-trained CNN models showed an increase in the model's accuracy in medical imaging	For all small datasets, data augmentation is required.
Li *et al*. (2023) [84]	Fundus images, (UBM, OCT)	Eye	CNN	Glaucoma classification through fine-tuning with ImageNet weights.	The proposed model achieved excellent performance under multimodal data in terms of Accuracy, Sensitivity, Specificity, and AUC.	The model is unable to properly learn the appropriate features of glaucoma due to the insufficient dataset without transfer learning.
Namachivayam and Puviarasan, (2023) [85]	MRI (KAGGLE)	Brain	Inceptionv3 and VGG19	Classification of AD, PD, Tumor, and Normal using feature extraction with the help of ImageNet	The Inceptionv3 along with Random Forest outperformed the other model in the comparison.	It did not compare with existing methods.
Ayana *et al*. (2023) [86]	Mammogram (DDSM)	Breast (Mass)	ViT-B	Classification of Breast mass *via* fine-tuning with ImageNet weights	Vision transformer-based transfer learning performed better than convolutional neural network-based transfer learning. Classification of diagnostic mammography.	Instead of using a dataset, the model experimented with a single image.
Majid *et al*. (2023) [87]	CT (PACS)	Kidney	ResNet-101 and DenseNet-121	Classification of tumors through fine-tuning with ImageNet	In this proposed method, the key features of computer-aided diagnosis (CAD) include increased precision, cost savings, and time effectiveness	Applicability to varied datasets still has to be studied. Examining other detection models in limited detail. It is still needed to investigate applicability to varied datasets.
Raza *et al*. (2023) [88]	MRI (ADNI)	Brain (Gray Matter)	Densenet-169	Classification of AD using ImageNet for fine-tuning	Even with a short dataset, the technique yielded better results by drastically lowering training time and minimizing overfitting.	The approach of transfer learning may result in negative transfer. Introductory training needs introductory challenges and homogeneous targets.
Kumar *et al*. (2023) [89]	MRI (ACRIN-DSC, CPTAC-GBM, ACRIN-FMISO)	Brain	ResNet-50, AlexNet, U-Net, and VGG-16	Classification of tumors through feature extraction using the given MRI dataset.	ResNet-50 considerably increased accuracy by 2%.	The VGG16 model displayed a 50% decline in performance.
Islam *et al*. (2023) [90]	MRI (Kaggle)	Brain	InceptionV3, VGG19, DenseNet121, and MobileNet	Prediction and classification of tumors through fine-tuning using ImageNet.	The MobileNet model yielded a high accuracy of 99.60%.	Only secondary datasets are used.
Alshahrani *et al*. (2023) [91]	Microscopy (Kaggle)	Bone	DenseNet121, EfficientNetB5, ResNet50, Xception, and MobileNetV2	Classification of BM cells *via* fine-tuning.	The DenseNet121 model obtained 99. 97% training and 97.01% testing performance.	No limitations were explicitly mentioned.
Abdulsahib *et al*. (2023) [92]	CT (Private)	Liver	VGG-16, ResNet-50, and MobileNetV2	Classification of liver cancer through fine-tuning using ImageNet.	The suggested approach achieves great accuracy in liver cancer categorization through TL.	A few samples have been wrongly classified. Limited sample size impairs generalization.
Elbashir *et al*. (2023) [93]	US	Liver	VGG16, VGG19, DenseNet, Inception, InceptionResNet, ResNet, and EfficientNet	Classification of liver cancer *via* fine-tuning and feature extraction using ImageNet.	The highest accuracy of 93% was attained upon optimizing the classification layer.	Patients and operators have a major impact on ultrasound performance.
Alhussan *et al*. (2023) [94]	Mammogram (DDSM)	Breast	AlexNet, GoogleNet, and VGG	Tumor Classification using fine-tuning along with ImageNet	Comparing the obtained results with existing methods, it was found that applying transfer learning and optimizing CNN for classification boosted classification accuracy.	The suggested optimization algorithm's complexity represents a potential drawback for the proposed strategy.
Thamizhvani (2024) [95]	MRI (ADNI)	Brain	Inception V3, GoogleNet, AlexNet, VGG16	Hippocampus segmentation and classification of AD through feature extraction using ImageNet weights	The suggested technique and VGG-16 are defined as more effective 3D segmentation and classification to accurately diagnose AD stages.	Restrictions are not made evident.
Zaabi *et al*. (2024) [96]	MRI (OASIS)	Brain	ViT-16 & ViT-32	Classification of AD through fine-tuning with ImageNet.	Transformers are used far more often than CNNs.	Few datasets are insufficient to train the model.
Mehmood *et al*. (2024) [97]	MRI (ADNI)	Brain (Gray Matter)	DSN (Deep Siamese Network), VGG-16	Detection and Classification of NC, MCI, and AD through fine-tuning along with ImageNet.	Great outcomes were achieved by the transfer learning-based approaches, which can aid in the creation of models for the early detection of AD.	No limitations are stated here.
El Houby (2024) [98]	X-ray	Chest (Lungs)	VGG-19 and EffcientNetB0	Classification of Lungs *via* fine-tuning and feature extraction along ImageNet	The model achieved the best binary classification accuracy of 95%. The model built using the Histeq version with VGG19 has the best achievable accuracy of 91% when trained on a segmented dataset.	Additional validations are necessary before implementing any system since matters about human life demand greater precision and precise experimentation.
Mammeri *et al*. (2024) [99]	CT (LIDC-IDRI)	Lungs	YOLO v7 and VGG-16	Classification of nodules through fine-tuning with ImageNet	Since the suggested method skips the lung segmentation and preprocessing methods that many other systems require, it saves a substantial amount of processing time and resources.	A data loss occurs while switching from 16 to 8 bits, potentially affecting system performance.
Liu *et al*. (2024) [100]	Microscopy (TG-GATE. WSI)	Kidney	ResNet-50	Classification of pathology *via* feature extraction with mouse glomerulus image weights.	The classification accuracy of the transfer learning method was 92.22%, surpassing the supervised learning model's 86.85% classification accuracy.	Due to data limitations, pretraining could only use images of the mouse and human glomerulus, making it unable to accurately assess the model's performance.
Legouis *et al*. (2024) [101]	Microscopy (TCGA, KIPAN, KIRC, and snRNAseq)	Kidney	K-CLIER	Classification of cell types *via* feature extraction by using K-CLIER image weights,	The study produced a distinct single-cell RNA-seq dataset derived from patients with acute kidney injury,	Signatures on a single cell are not standardized. K-CLIER is limited to use with bulk RNA sequencing.
Rajput *et al*. (2024) [102]	MRI	Brain	VGG19, Inception-v3, and ResNet50	Classification of tumor via feature extraction from ImageNet.	The model successfully mitigates the overfitting issue.	Limitations are not specifically mentioned in the paper.
Singh and Lobiyal (2024) [103]	MRI (Figshare)	Brain	Xception, Inceptionv3, VGG19, and DenseNet201	Classification of tumors *via* fine-tuning using ImageNet	DTL outperforms classical ML, scoring over 96%.	High processing demands challenge the implementation of low-power devices.
Khaliki & Başarslan, (2024) [104]	MRI (Kaggle)	Brain	Inception-V3, EfcientNetB4, VGG16, VGG19	Classification of tumors through feature extraction along with ImageNet.	The suggested work uses the VGG16 model to classify brain tumors with a high accuracy of 98%.	The dataset was employed without any cropping or rotation.
Raina *et al*. (2024) [105]	MRI, X-ray	Brain, Chest	VGG 16	Classification of tumors and diagnosing pneumonia by fine-tuning using ImageNet	The model's accuracy was increased to 93.6% by optimizing the VGG-16 model.	The model shows a small amount of overfitting.
Al-Otaibi *et al*. (2024) [106]	MRI (Kaggle)	Brain (Lesions)	VGG 16	Classification *via* feature extraction using ImageNet	Diagnostic accuracy is increased by efficient feature extraction from MRI images.	The cost of computing is high for training.
Mathivanan *et al*. (2024) [107]	MRI (Kaggle)	Brain	ResNet152, VGG19, DenseNet169, and MobileNetv3	Classification by feature extraction with ImageNet weights.	MobileNetv3 integrates deep learning and transfer learning to diagnose brain tumors with 99. 75% accuracy.	The model's functionality in various scenarios was not examined. Model usage and training costs were not taken into account.
Wang (2024) [108]	MRI (BraTS)	Brain	EfficientNet B3	Segmentation and classification *via* feature extraction by ImageNet	The model successfully distinguishes between surrounding tissues and tumor cores in a variety of datasets.	With rare tumor types, performance may deteriorate. The model complexity and requirement for Computation.
Remya *et al*. (2024) [109]	Dermatoscopy (HAM10000)	Skin	EfficientNet-B0	Classification of lesions is done by fine-tuning and feature extraction with ImageNet.	The proposed work detects skin lesions with 99% accuracy.	Single-modality images limit diagnostic accuracy.
Khaled *et al*. (2024) [110]	Mammogram (INbreast)	Breast	VGG16, ResNet, EfficientNet, DenseNet, Xception	Classification of cancer *via* fine-tuning using ImageNet	Addresses imbalanced data to improve diagnostic precision and enhanced sensitivity due to incremental transfer learning of image characteristics.	Risk of overfitting because of unbalanced data.
Devi *et al*. (2024) [111]	Mammogram, MRI	Breast	ResNet	Classification of cancer *via* fine-tuning and feature extraction with Breast Cancer dataset	Integrating federated learning (FL) improves data security and privacy, allowing numerous healthcare organizations to jointly build a global framework without exchanging unprocessed information.	Resolving domain shift concerns
Albalawi *et al*. (2024) [112]	MRI (figshare, SARTAJ, and Br35H)	Brain	VGG-16	Classification of tumors through feature extraction and fine-tuning by the use of ImageNet	The suggested study improves the accuracy of brain tumor classification with MRI scans, obtaining an overall precision of 98%.	Model training requires significant computer resources. Limited generalization across different patient groups.

**Table 3:** Summary of deep transfer learning-based disease detection techniques for medical image processing.

**References**	**Imaging Modality used**	**Focused Regions**	**Pre-trained model adopted**	**Tasks Undertaken**	**Significance**	**Limitations**
Yap *et al*. (2018) [113]	US (Didactic media file, UDIAT)	Breast	FCN-AlexNet	Lesion Detection by feature extraction with ImageNet	For the Didactic media file, the Transfer Learning FCN-AlexNet approach produced the greatest results out of all the approaches covered in this research.	The training process, which takes a long period and needs a representative sample of typical images, is the primary downside. In clinical settings, obtaining these images during an ultrasonic examination is not standard procedure.
Khalifa *et al*. (2019) [114]	FUNDUS (APTOS)	Eye (Retina)	AlexNet, Res-Net18, SqueezeNet, GoogleNet, VGG16, and VGG19	Detection of Diabetic Retinopathy (DR) *via* feature extraction along with ImageNet	The AlexNet model has the greatest testing accuracy (97.9%) with reduced training time and computational complexity by the minimum number of layers.	No clear restrictions are made.
Lu *et al*. (2019) [115]	MRI (Private)	Brain	AlexNet	Detection of tumors by feature extraction using ImageNet	The transfer learning approach increases training effectiveness to hasten convergence.	The emphasis on specific brain illnesses is unknown.
Si Xiaopeng *et al*. (2020) [116]	diffusion MRI	Brain (White Matter Fibers)	CNN, Inception_v3, Inception_v4, Inception_resnet_v2	Detection of white matter changes (JME) through feature extraction with ImageNet	This technique might improve the JME assessment of diagnostics.	Limited data size has affected accuracy.
Gangwar and Ravi (2021) [117]	Fundus photography (Messidor-1 and APTOS)	Eye	Inception-ResNet-v2	Detection of DR is done through feature extraction and fine-tuning using ImageNet.	The model achieved 72. 33% on Messidor-1 and 82. 18% on APTOS 2019.	Limited testing of model generalizability to various datasets and clinical settings.
Harsono *et al*. (2022) [118]	CT (LIDC-IDRI, Moscow Private Datasets)	Lungs	Inception 3D	Nodule detection and classification *via* inflating with ImageNet weights	Optimizing detection performance and reducing training time can be achieved using weight transfer learning using natural image datasets.	No limitations are mentioned.
Islam *et al*. (2022) [119]	CT (Urogram) (PACS)	Kidney	ResNet50, VGG16, and Inception v3	Detection of cysts, tumors, and stones through feature extraction using ImageNet	The VGG16-based model and the Swin transformer perform better than all the other models in a shorter training period.	There are no restrictions stated.
Jabbar *et al*. (2022) [120]	Fundus images (Kaggle-EyePACS)	Eye	VGG 16	Detection and Classification of DR by feature extraction using ImageNet.	This framework outperforms advanced methods in accuracy on benchmark datasets.	Classification accuracy is impacted by an unbalanced dataset distribution.
Altun *et al*. (2023) [121]	Digital Camera imaging (MSLD) (KAGGLE)	Skin	MobileNetV3, EfficientNetV2, ResNET50, Vgg19, DenseNet121, and Xception	Monkeypox Detection using fine-tuning with ImageNet	The model MobileNetV3-s attained the best accuracy rate.	The formal medical community does not classify the image files used to identify monkeypox disease. Health organizations have not yet released an official dataset.
Chui *et al*. (2023) [122]	CT (NSCLC-Radiomics, Genomics, SPIE-AAPM Lung CT Challenge)	Lungs	CNN	Cancer detection *via* fine-tuning with the help of ImageNet	Compared to related efforts, it greatly enhances the lung cancer detection model's performance.	The suggested strategy makes the optimization algorithm more difficult.
Bhattacharjee *et al*. (2023) [123]	CT (IQ-OTH NCCD (lungs), NCTS(Kidney))	Kidney and Lungs	Xception	Detection of cancer *via* fine-tuning along with ImageNet.	For early detection, the improved Xception model performs better than the original.	There is not enough training data for the proposed MNoB model. Context extraction is limited by the two dimensions of the proposed models.
Terzi and Azginoglu (2023) [124]	MRI (MS COCO, BraTS 2020, Gazi Brains 2020)	Brain	Mask R-CNN and YOLOv8	Detection of tumors *via* fine-tuning along with ImageNet weights.	The study found that in-domain transfer learning beats pre-trained models on natural images for detecting brain tumors.	Only the BraTS dataset was used as a targeted transfer learning source.
Pandey and Bhandari (2024) [125]	MRI (BraTS21)	Brain	AlexNet, ResNet-50, MobileNet, GoogleNet, YOLOv3, YOLOv4, YOLOv5	Detection of tumor *via* fine-tuning with ImageNet	Out of all the versions, the YOLOv5x model had the highest accuracy.	The YOLOv5 model can achieve state-of-the-art results for object detection, but sometimes it has trouble identifying very small objects in the test image.
Ashafuddula and Islam (2024) [126]	MRI (Kaggle)	Brain	VGG 16	Detection and Classification of tumors *via* feature extraction with ImageNet.	The pre-trained VGG-16 model boosts feature extraction and classification efficiency.	Constrained by hardware limitations for larger datasets

## LIST OF ABBREVIATIONS

ADNI: Alzheimer’s Disease Neuroimaging Initiative
AI: Artificial Intelligence
ALL: Acute Lymphocytic Leukemia
AML: leukemia Acute Myeloid Leukemia
APTOS: Asia Pacific Tele-Ophthalmology Society
AUC: Area Under the Curve
BI-RADS: The Breast Imaging-Reporting and Data System
BraTS: Brain Tumor Segmentation Challenge
CBTN: Children's Brain Tumor Network
CE-MRI: Contrast-Enhanced Magnetic Resonance Images
CHUAC: University Hospital Complex of A Coruña
CKD: Chronic Kidney Disease
CLL: Chronic Lymphocytic Leukemia
CML: Chronic Myeloid Leukemia
CNN: Convolutional Neural Network
CT: Computed Tomography
DBT: Digital Breast Tomosynthesis
DCE-CT: Dynamic Contrast-Enhanced Computed Tomography
DDSM: Digital Database for Screening Mammography
DL: Deep Learning
DM: Digital Mammography
DR: Diabetic Retinopathy
DTL: Deep Transfer Learning
EUS: Endoscopic Ultrasound
GE-MRI: General Electric Magnetic Resonance Imaging machine
GI: Gastro-Intestinal
GPT: Generative Pre-trained Transformer
HuBMAP: Human BioMolecular Atlas Program
IDRI: Image Database Resource Initiative
ILSVRC: ImageNet Large Scale Visual Recognition Challenge
IQ-OTH/ NCCD: Iraq-Oncology Teaching Hospital/National Center for Cancer Diseases
ISBI: International Symposium on Biomedical Imaging
ISIC: International Skin Imaging Collaboration
ISLES: Ischemic Stroke Lesion Segmentation
JME: Juvenile Myoclonic Epilepsy
JSRT: Japanese Society of Radiological Technology
K-CLIER: kidney-specific cell-level information ExtractoR
KIPAN: Pan-kidney
KIRC: Kidney renal clear cell carcinoma
KiTS: Kidney Tumor Segmentation Challenge
LIDC: Lung Image Database Consortium
LiTS: Liver Tumor Segmentation Challenge
MIL: Multiple instance learning
MRI: Magnetic Resonance Imaging
MS COCO: Microsoft Common Objects in Context
MSD: Medical Segmentation Decathlon Challenge
MSLD: Monkeypox Skin Lesion Dataset
NLP: Natural Language Programming
NTU: National Taiwan University
OASBUD: Open Access Series of Breast Ultrasonic Data
OCT: Optical Coherence Tomography
ODIR: Ocular Disease Intelligent Recognition
PACS: Picture Archiving and Communication System
PET: Positron Emission Tomography
SARS-CoV-2: Severe Acute Respiratory Syndrome Coronavirus 2
SFM: Screen-Film Mammography
SPECT: Single Photon Emission Computed Tomography
TCGA: The Cancer Genome Atlas
TCIA: The Cancer Imaging Archive
TL: Transfer learning
UBM: Ultrasound Biomicroscopy
US: Ultrasound Scans
VGG: Visual Geometry Group
WMH: White Matter Hyperintensities
WSIs: Whole-Slide Images

## References

[1] Rahim R., Sabillah B.M., Sukmawati, Suhardianto, Cakrawijaya M.H., Ginting W.A. (2019). Diagnosis in medical imaging.. Int. J. Eng. Adv. Technol..

[2] Iman M., Arabnia H.R., Branchinst R.M., Arabnia H.R., Ferens K., de la Fuente D., Kozerenko E.B., Olivas Varela J.A., Tinetti F.G. (2021). Pathways to artificial general intelligence: A brief overview of developments and ethical issues *via* artificial intelligence, machine learning, deep learning, and data science.. Advances in Artificial Intelligence and Applied Cognitive Computing.

[3] Zhuang F., Qi Z., Duan K., Xi D., Zhu Y., Zhu H., Xiong H., He Q. (2021). A comprehensive survey on transfer learning.. Proc. IEEE.

[4] Farahani A., Voghoei S., Rasheed K., Arabnia H.R. A brief review of domain adaptation.. Advances in Data Science and Information Engineering. Transactions on Computational Science and Computational Intelligence..

[5] Niu S., Liu Y., Wang J., Song H. (2020). A decade survey of transfer learning (2010–2020).. IEEE Trans. Artif. Intell..

[6] Pan S.J., Yang Q. (2010). A survey on transfer learning.. IEEE Trans. Knowl. Data Eng..

[7] Carlucci F.M., Porzi L., Caputo B., Ricci E., Bulò S.R. AutoDIAL: Automatic domain alignment layers.. 2017 IEEE International Conference on Computer Vision (ICCV).

[8] Iman M., Arabnia H.R., Rasheed K. (2023). A review of deep transfer learning and recent advancements.. Technol..

[9] Saha S., Ahmad T. (2021). Federated transfer learning: Concept and applications.. Intell. Artif. (Int. J. AIxIA).

[10] Sarker I.H. (2021). Deep learning: A comprehensive overview on techniques, taxonomy, applications and research directions.. SN Comput Sci.

[11] Dey R., Balabantaray R.C., Samanta S. (2022). Recognition of handwritten characters from Devanagari, Bangla, and Odia languages using transfer-learning-based VGG-16 networks.. Imaging Sci. J..

[12] Islam M.M. (2024). The impact of transfer learning on AI performance across domains.. J. Artif. Intell. Gen. Sci..

[13] (2021). About ImageNet.. https://www.image-net.org/about.php.

[14] Saxena P., Goyal A. (2022). Computer-assisted grading of follicular lymphoma: A classification based on SVM, machine learning, and transfer learning approaches.. Imaging Sci. J..

[15] Deng J., Dong W., Socher R., Li L.J., Li K., Fei-Fei L. ImageNet: A large-scale hierarchical image database.. 2009 IEEE Conference on Computer Vision and Pattern Recognition.

[16] Bozinovski S. (2020). Reminder of the first paper on transfer learning in neural networks, 1976.. Informatica.

[17] Pratt L.Y. Discriminability-based transfer between neural networks.. NIPS'92: Proceedings of the 6th International Conference on Neural Information Processing Systems.

[18] Donahue J., Jia Y., Vinyals O., Hoffman J., Zhang N., Tzeng E., Darrell T. (2014). DeCAF: A deep convolutional activation feature for generic visual recognition.. arXiv.

[19] Liu S., Deng W. Very deep convolutional neural network based image classification using small training sample size.. 2015 3rd IAPR Asian Conference on Pattern Recognition (ACPR).

[20] Tan C., Sun F., Kong T., Zhang W., Yang C., Liu C. A survey on deep transfer learning.. Artificial Neural Networks and Machine Learning – ICANN 2018. ICANN 2018. Lecture Notes in Computer Science.

[21] Chang H.S., Fu M.C., Hu J., Marcus S.I. (2016). Google DeepMind's AlphaGo: Operations research's unheralded role in the path-breaking achievement.. OR/MS Today.

[22] Rusu A.A., Rabinowitz N.C., Desjardins G., Soyer H., Kirkpatrick J., Kavukcuoglu K., Pascanu R., Hadsell R. (2016). Progressive neural networks.. arXiv.

[23] Sistaninejhad B., Rasi H., Nayeri P. (2023). A review paper about deep learning for medical image analysis.. Comput Math Methods Med.

[24] Morid M.A., Borjali A., Del Fiol G. (2021). A scoping review of transfer learning research on medical image analysis using ImageNet.. Comput Biol Med.

[25] Sharma S., Mehra R. (2020). Conventional machine learning and deep learning approach for multi-classification of breast cancer histopathology images—A comparative insight.. J. Digit. Imaging.

[26] Litjens G., Kooi T., Ehteshami Bejnordi B., Setio A.A.A., Ciompi F., Ghafoorian M., van der Laak J.A.W.M., van Ginneken B., Sánchez C.I. (2017). A survey on deep learning in medical image analysis.. Med Image Anal.

[27] Xu Y., Géraud T., Bloch I. From neonatal to adult brain MR image segmentation in a few seconds using 3D-like fully convolutional network and transfer learning.. 2017 IEEE International Conference on Image Processing (ICIP).

[28] Xu Y., Géraud T., Puybareau É., Bloch I., Chazalon J. White matter hyperintensities segmentation in a few seconds using fully convolutional network and transfer learning.. Brainlesion: Glioma, Multiple Sclerosis, Stroke and Traumatic Brain Injuries. BrainLes 2017. Lecture Notes in Computer Science.

[29] Oliveira H., Dos Santos J. Deep transfer learning for segmentation of anatomical structures in chest radiographs.. 2018 31st SIBGRAPI Conference on Graphics, Patterns and Images (SIBGRAPI).

[30] Hamed Mozaffari M., Lee W.S. (2019). Domain adaptation for ultrasound tongue contour extraction using transfer learning: A deep learning approach.. J. Acoust. Soc. Am..

[31] Amin J., Sharif M., Yasmin M., Saba T., Anjum M.A., Fernandes S.L. (2019). A new approach for brain tumor segmentation and classification based on score level fusion using transfer learning.. J. Med. Syst..

[32] Chen S., Ma K., Zheng Y. (2019). Med3D: Transfer learning for 3D medical image analysis.. arXiv.

[33] Soudani A., Barhoumi W. (2019). An image-based segmentation recommender using crowdsourcing and transfer learning for skin lesion extraction.. Expert Syst. Appl..

[34] Puybareau É., Zhao Z., Khoudli Y., Carlinet E., Xu Y., Lacotte J., Géraud T. Left atrial segmentation in a few seconds using fully convolutional network and transfer learning.. Statistical Atlases and Computational Models of the Heart. Atrial Segmentation and LV Quantification Challenges. STACOM 2018. Lecture Notes in Computer Science.

[35] Lee J., Nishikawa R.M. (2020). Cross-organ, cross-modality transfer learning: Feasibility study for segmentation and classification.. IEEE Access.

[36] Al Nazi Z., Abir T.A. Automatic skin lesion segmentation and melanoma detection: Transfer learning approach with U-Net and DCNN-SVM.. Proceedings of International Joint Conference on Computational Intelligence. Algorithms for Intelligent Systems.

[37] Vu C.C., Siddiqui Z.A., Zamdborg L., Thompson A.B., Quinn T.J., Castillo E., Guerrero T.M. (2020). Deep convolutional neural networks for automatic segmentation of thoracic organs-at-risk in radiation oncology – use of non-domain transfer learning.. J. Appl. Clin. Med. Phys..

[38] Vidal P.L., de Moura J., Novo J., Ortega M. (2021). Multi-stage transfer learning for lung segmentation using portable X-ray devices for patients with COVID-19.. Expert Syst. Appl..

[39] Cheng D., Lam E.Y. (2021). Transfer learning U-net deep learning for lung ultrasound segmentation.. arXiv.

[40] Karimi D., Vasylechko S., Gholipour A. Convolution-free medical image segmentation using transformers.. Medical Image Computing and Computer Assisted Intervention – MICCAI 2021. MICCAI 2021. Lecture Notes in Computer Science.

[41] Liu J., Dong B., Wang S., Cui H., Fan D.P., Ma J., Chen G. (2021). COVID-19 lung infection segmentation with a novel two-stage cross-domain transfer learning framework.. Med. Image Anal..

[42] Hsiao C.-H., Tsai M.-C., Lin F. Y.-S., Lin P.-C., Yang F.-J., Yang S.-Y., Wang S.-Y., Liu P.-R., Huang Y. Automatic kidney volume estimation system using transfer learning techniques.. Advanced Information Networking and Applications. AINA 2021. Lecture Notes in Networks and Systems..

[43] Ankenbrand M.J., Lohr D., Schlötelburg W., Reiter T., Wech T., Schreiber L.M. (2021). Deep learning-based cardiac cine segmentation: Transfer learning application to 7T ultrahigh-field MRI.. Magn. Reson. Med..

[44] Zhu X.L., Shen H.B., Sun H., Duan L.X., Xu Y.Y. (2022). Improving segmentation and classification of renal tumors in small sample 3D CT images using transfer learning with convolutional neural networks.. Int. J. CARS.

[45] Araújo R.L., Araújo F.H.D.d., Silva R.R.V.e. (2022). Automatic segmentation of melanoma skin cancer using transfer learning and fine-tuning.. Multimed Syst.

[46] Maiello L., Ball L., Micali M., Iannuzzi F., Scherf N., Hoffmann R.T., Gama de Abreu M., Pelosi P., Huhle R. (2022). Automatic lung segmentation and quantification of aeration in computed tomography of the chest using 3D transfer learning.. Front. Physiol..

[47] Ambesange S., Annappa B., Koolagudi S.G. (2023). Simulating federated transfer learning for lung segmentation using modified Unet model.. Procedia Comput. Sci..

[48] Riaz Z., Khan B., Abdullah S., Khan S., Islam M.S. (2023). Lung tumor image segmentation from computer tomography images using MobileNetV2 and transfer learning.. Bioengineering.

[49] Boyd A., Ye Z., Prabhu S., Tjong M.C., Zha Y., Zapaishchykova A., Vajapeyam S., Hayat H., Chopra R., Liu K.X., Nabavidazeh A., Resnick A., Mueller S., Haas-Kogan D., Aerts H.J.W.L., Poussaint T., Kann B.H. (2023). Expert-level pediatric brain tumor segmentation in a limited data scenario with stepwise transfer learning.. medRxiv.

[50] Sharma N., Gupta S., Koundal D., Alyami S., Alshahrani H., Asiri Y., Shaikh A. (2023). U-net model with transfer learning model as a backbone for segmentation of gastrointestinal tract.. Bioengineering.

[51] Ali S., Agrawal J. (2023). Automated segmentation of brain tumour images using deep learning-based model VGG19 and ResNet 101.. Multimedia Tools Appl..

[52] Ren T., Honey E., Rebala H., Sharma A., Chopra A., Kurt M. An optimization framework for processing and transfer learning for the brain tumor segmentation.. Brain Tumor Segmentation, and Cross-Modality Domain Adaptation for Medical Image Segmentation. crossMoDA BraTS 2023 2023. Lecture Notes in Computer Science.

[53] Srivastava G.R., Gera P., Rani R., Jaiswal G., Sharma A. (2025). A novel method for glioma segmentation and classification on pre-operative MRI scans using 3D U-Nets and transfer learning.. Multimed Tools Appl.

[54] Oostrom M., Muniak M.A., Eichler West R.M., Akers S., Pande P., Obiri M., Wang W., Bowyer K., Wu Z., Bramer L.M., Mao T., Webb-Robertson B.J.M. (2024). Fine-tuning TrailMap: The utility of transfer learning to improve the performance of deep learning in axon segmentation of light-sheet microscopy images.. PLoS One.

[55] Basha N.K., Ananth C., Muthukumaran K., Sudhamsu G., Mittal V., Gared F. (2024). Mask region-based convolutional neural network and VGG-16 inspired brain tumor segmentation.. Sci. Rep..

[56] Clark B., Hardcastle N., Johnston L.A., Korte J. (2024). Transfer learning for auto-segmentation of 17 organs-at-risk in the head and neck: Bridging the gap between institutional and public datasets.. Med. Phys..

[57] Aung T.M.M., Khan A.A. (2025). Enhanced U-net with attention mechanisms for improved feature representation in lung nodule segmentation.. Curr. Med. Imaging.

[58] Samala R.K., Chan H.P., Hadjiiski L.M., Helvie M.A., Cha K.H., Richter C.D. (2017). Multi-task transfer learning deep convolutional neural network: Application to computer-aided diagnosis of breast cancer on mammograms.. Phys. Med. Biol..

[59] Meng D., Zhang L., Cao G., Cao W., Zhang G., Hu B. (2017). Liver fibrosis classification based on transfer learning and FCNet for ultrasound images.. IEEE Access.

[60] Li H., Parikh N.A., He L. (2018). A novel transfer learning approach to enhance deep neural network classification of brain functional connectomes.. Front. Neurosci..

[61] Byra M., Galperin M., Ojeda-Fournier H., Olson L., O’Boyle M., Comstock C., Andre M. (2019). Breast mass classification in sonography with transfer learning using a deep convolutional neural network and color conversion.. Med. Phys..

[62] Hijab A., Rushdi M.A., Gomaa M.M., Eldeib A. Breast cancer classification in ultrasound images using transfer learning.. 2019 Fifth International Conference on Advances in Biomedical Engineering (ICABME).

[63] Samala R.K., Chan H-P., Hadjiiski L., Helvie M.A., Richter C.D., Cha K.H. (2019). Breast cancer diagnosis in digital breast tomosynthesis: Effects of training sample size on multi-stage transfer learning using deep neural nets.. IEEE Trans. Med. Imaging.

[64] Swati Z.N.K., Zhao Q., Kabir M., Ali F., Ali Z., Ahmed S., Lu J. (2019). Brain tumor classification for MR images using transfer learning and fine-tuning.. Comput. Med. Imaging Graph..

[65] Talo M., Baloglu U.B., Yıldırım Ö., Rajendra Acharya U. (2019). Application of deep transfer learning for automated brain abnormality classification using MR images.. Cogn. Syst. Res..

[66] Deepak S., Ameer P.M. (2019). Brain tumor classification using deep CNN features *via* transfer learning.. Comput. Biol. Med..

[67] Yamada A., Oyama K., Fujita S., Yoshizawa E., Ichinohe F., Komatsu D., Fujinaga Y. (2019). Dynamic contrast-enhanced computed tomography diagnosis of primary liver cancers using transfer learning of pretrained convolutional neural networks: Is registration of multiphasic images necessary?. Int. J. CARS.

[68] Loey M., Smarandache F., Khalifa N.E.M. (2020). Within the lack of chest COVID-19 X-ray dataset: A novel detection model based on GAN and deep transfer learning.. Symmetry.

[69] Mehrotra R., Ansari M.A., Agrawal R., Anand R.S. (2020). A transfer learning approach for AI-based classification of brain tumors.. Mach. Learn. Appl..

[70] Rehman A., Naz S., Razzak M.I., Akram F., Imran M. (2020). A deep learning-based framework for automatic brain tumors classification using transfer learning.. Circuits Syst. Signal Process..

[71] Kaur T., Gandhi T.K. (2020). Deep convolutional neural networks with transfer learning for automated brain image classification.. Mach Vis Appl.

[72] Loey M., Naman M., Zayed H. (2020). Deep transfer learning in diagnosing leukemia in blood cells.. Computers.

[73] Rodrigues D.A., Ivo R.F., Satapathy S.C., Wang S., Hemanth J., Filho P.P.R. (2020). A new approach for classification skin lesion based on transfer learning, deep learning, and IoT system.. Pattern Recognit. Lett..

[74] Sun C., Xu A., Liu D., Xiong Z., Zhao F., Ding W. (2020). Deep learning-based classification of liver cancer histopathology images using only global labels.. IEEE J. Biomed. Health Inform..

[75] Gu Y., Ge Z., Bonnington C.P., Zhou J. (2020). Progressive transfer learning and adversarial domain adaptation for cross-domain skin disease classification.. IEEE J. Biomed. Health Inform..

[76] Ayana G., Dese K., Choe S.W. (2021). Transfer learning in breast cancer diagnoses *via* ultrasound imaging.. Cancers.

[77] Gour N., Khanna P. (2021). Multi-class multi-label ophthalmological disease detection using transfer learning based convolutional neural network.. Biomed. Signal Process. Control.

[78] Aftab M.O., Javed Awan M., Khalid S., Javed R., Shabir H. Executing spark BigDL for leukemia detection from microscopic images using transfer learning.. 2021 1st International Conference on Artificial Intelligence and Data Analytics (CAIDA).

[79] Aswathi T., Swapna T.R., Padmavathi S. (2021). Transfer Learning approach for grading of Diabetic Retinopathy.. J. Phys. Conf. Ser..

[80] Islam M.M., Islam M.Z., Asraf A., Al-Rakhami M.S., Ding W., Sodhro A.H. (2022). Diagnosis of COVID-19 from X-rays using combined CNN-RNN architecture with transfer learning.. BenchCouncil Trans. Benchm. Stand. Eval..

[81] Lee H.C., Aqil A.F. (2022). Combination of transfer learning methods for kidney glomeruli image classification.. Appl. Sci..

[82] Hasan M.K., Elahi M.T.E., Alam M.A., Jawad M.T., Martí R. (2022). DermoExpert: Skin lesion classification using a hybrid convolutional neural network through segmentation, transfer learning, and augmentation.. Inform. Med. Unlocked.

[83] Khaled M., Gaceb D., Touazi F., Otsmane A., Boutoutaou F. Progressive and combined deep transfer learning for pneumonia diagnosis in chest X-ray images.. IDDM-2022: 5th International Conference on Informatics & Data-Driven Medicine.

[84] Li Y., Han Y., Li Z., Zhong Y., Guo Z. (2023). A transfer learning-based multimodal neural network combining metadata and multiple medical images for glaucoma type diagnosis.. Sci. Rep..

[85] Namachivayam A., Puviarasan N. (2023). Computerised brain tumours classification using MRI images.. Int J Intell Syst Appl Eng.

[86] Ayana G., Dese K., Dereje Y., Kebede Y., Barki H., Amdissa D., Husen N., Mulugeta F., Habtamu B., Choe S.W. (2023). Vision-transformer-based transfer learning for mammogram classification.. Diagnostics.

[87] Majid M., Gulzar Y., Ayoub S., Khan F., Reegu F.A., Mir M.S., Jaziri W., Soomro A.B. (2023). Enhanced transfer learning strategies for effective kidney tumor classification with CT imaging.. Int. J. Adv. Comput. Sci. Appl..

[88] Raza N., Naseer A., Tamoor M., Zafar K. (2023). Alzheimer disease classification through transfer learning approach.. Diagnostics.

[89] Kumar S., Choudhary S., Jain A., Singh K., Ahmadian A., Bajuri M.Y. (2023). Brain tumor classification using deep neural network and transfer learning.. Brain Topogr..

[90] Islam M.M., Barua P., Rahman M., Ahammed T., Akter L., Uddin J. (2023). Transfer learning architectures with fine-tuning for brain tumor classification using magnetic resonance imaging.. Healthc. Anal..

[91] Alshahrani H., Sharma G., Anand V., Gupta S., Sulaiman A., Elmagzoub M.A., Reshan M.S.A., Shaikh A., Azar A.T. (2023). An intelligent attention-based transfer learning model for accurate differentiation of bone marrow stains to diagnose hematological disorder.. Life.

[92] Abdulsahib F.I., Al-Khateeb B., Kóczy L.T., Nagy S. (2023). A transfer learning approach for the classification of liver cancer.. J. Intell. Syst..

[93] Elbashir M.K., Mahmoud A., Mostafa A.M., Hamouda E., Alruily M., Alotaibi S.M., Shabana H., Ezz M. (2023). A transfer learning approach based on ultrasound images for liver cancer detection.. Comput. Mater. Continua.

[94] Alhussan A.A., Abdelhamid A.A., Towfek S.K., Ibrahim A., Abualigah L., Khodadadi N., Khafaga D.S., Al-Otaibi S., Ahmed A.E. (2023). Classification of breast cancer using transfer learning and advanced Al-Biruni Earth radius optimization.. Biomimetics.

[95] Thamizhvani T.R., Hemalatha R.J. (2024). Classification of the different stages of Alzheimer’s disease using 3D transfer learning networks.. Res Sq.

[96] Zaabi M., Khedher M.I., El-Yacoubi M.A. Improving Alzheimer’s diagnosis using vision transformers and transfer learning.. 2024 16th International Conference on Human System Interaction (HSI).

[97] Mehmood A., Zheng Z., Khan R., Al Smadi A., Shahid F., Iqbal S., Alsmadi M.K., Ghadi Y.Y., Shah S.A., Ibrahim M.M. (2024). Enhancing mild cognitive impairment detection through efficient magnetic resonance image analysis.. Comput. Mater. Continua.

[98] El Houby E.M.F. (2024). COVID‑19 detection from chest X-ray images using transfer learning.. Sci. Rep..

[99] Mammeri S., Amroune M., Haouam M.Y., Bendib I., Corrêa Silva A. (2023). Early detection and diagnosis of lung cancer using YOLO v7, and transfer learning.. Multimedia Tools Appl..

[100] Liu X., Zhu X., Tian X., Iwasaki T., Sato A., Kazama J.J. (2024). Renal pathological image classification based on contrastive and transfer learning.. Electronics.

[101] Legouis D., Rinaldi A., Malpetti D., Arnoux G., Verissimo T., Faivre A., Mangili F., Rinaldi A., Ruinelli L., Pugin J., Moll S., Clivio L., Bolis M., de Seigneux S., Azzimonti L., Cippà P.E. (2024). A transfer learning framework to elucidate the clinical relevance of altered proximal tubule cell states in kidney disease.. iScience.

[102] Rajput I.S., Gupta A., Jain V., Tyagi S. (2023). A transfer learning-based brain tumor classification using magnetic resonance images.. Multimedia Tools Appl..

[103] Singh Y.P., Lobiyal D.K. (2023). A comparative analysis and classification of cancerous brain tumors detection based on classical machine learning and deep transfer learning models.. Multimedia Tools Appl..

[104] Khaliki M.Z., Başarslan M.S. (2024). Brain tumor detection from images and comparison with transfer learning methods and 3-layer CNN.. Sci. Rep..

[105] Raina D., Dawange A., Bandha T., Kaur A., Wasekar R., Verma K., Verma S., Dhingra K. (2024). Convoluted neural network and transfer learning algorithm for improved brain tumor classifications in MRI.. J Knowl Learn Sci Technol.

[106] Al-Otaibi S., Rehman A., Raza A., Alyami J., Saba T. (2024). CVG-net: Novel transfer learning based deep features for diagnosis of brain tumors using MRI scans.. PeerJ Comput. Sci..

[107] Mathivanan S.K., Sonaimuthu S., Murugesan S., Rajadurai H., Shivahare B.D., Shah M.A. (2024). Employing deep learning and transfer learning for accurate brain tumor detection.. Sci. Rep..

[108] Wang S. (2024). The future of brain tumor diagnosis: CNN And transfer learning innovations.. Mathematics, Statistics, and Computer Science Honors Projects.

[109] Remya S., Anjali T., Sugumaran V. (2024). A novel transfer learning framework for multimodal skin lesion analysis.. IEEE Access.

[110] Khaled M., Touazi F., Gaceb D. (2025). Improving breast cancer diagnosis in mammograms with progressive transfer learning and ensemble deep learning.. Arab J Sci Eng.

[111] S S., Dharani Devi G., v R., Jeyalakshmi J. (2024). Privacy-preserving breast cancer classification: A federated transfer learning approach.. J. Imaging Inform. Med..

[112] Albalawi E., T R M., Thakur A., Kumar V.V., Gupta M., Khan S.B., Almusharraf A. (2024). Integrated approach of federated learning with transfer learning for classification and diagnosis of brain tumor.. BMC Med. Imaging.

[113] Yap M.H., Pons G., Martí J., Ganau S., Sentís M., Zwiggelaar R., Davison A.K., Martí R., Pons G., Marti J., Ganau S., Sentis M., Zwiggelaar R., Davison A.K., Marti R., Moi Hoon Yap (2018). Automated breast ultrasound lesions detection using convolutional neural networks.. IEEE J. Biomed. Health Inform..

[114] Khalifa N., Loey M., Taha M., Mohamed H. (2019). Deep transfer learning models for medical diabetic retinopathy detection.. Acta Inform. Med..

[115] Lu S., Lu Z., Zhang Y.D. (2019). Pathological brain detection based on AlexNet and transfer learning.. J. Comput. Sci..

[116] Si X., Zhang X., Zhou Y., Sun Y., Jin W., Yin S., Zhao X., Li Q., Ming D. Automated detection of juvenile myoclonic epilepsy using CNN based transfer learning in diffusion MRI.. 2020 42nd Annual International Conference of the IEEE Engineering in Medicine & Biology Society (EMBC).

[117] Gangwar A.K., Ravi V. Diabetic retinopathy detection using transfer learning and deep learning.. Evolution in Computational Intelligence. Advances in Intelligent Systems and Computing..

[118] Harsono I.W., Liawatimena S., Cenggoro T.W. (2022). Lung nodule detection and classification from thorax CT-scan using RetinaNet with transfer learning.. J King Saud Univ Comput Inf Sci.

[119] Islam M.N., Hasan M., Hossain M.K., Alam M.G.R., Uddin M.Z., Soylu A. (2022). Vision transformer and explainable transfer learning models for auto detection of kidney cyst, stone and tumor from CT-radiography.. Sci. Rep..

[120] Jabbar M.K., Yan J., Xu H., Ur Rehman Z., Jabbar A. (2022). Transfer learning-based model for diabetic retinopathy diagnosis using retinal images.. Brain Sci..

[121] Altun M., Gürüler H., Özkaraca O., Khan F., Khan J., Lee Y. (2023). Monkeypox detection using CNN with transfer learning.. Sensors.

[122] Chui K.T., Gupta B.B., Jhaveri R.H., Chi H.R., Arya V., Almomani A., Nauman A. (2023). Multiround transfer learning and modified generative adversarial network for lung cancer detection.. Int. J. Intell. Syst..

[123] Bhattacharjee A., Rabea S., Bhattacharjee A., Elkaeed E.B., Murugan R., Selim H.M.R.M., Sahu R.K., Shazly G.A., Salem Bekhit M.M. (2023). A multi-class deep learning model for early lung cancer and chronic kidney disease detection using computed tomography images.. Front. Oncol..

[124] Terzi D.S., Azginoglu N. (2023). In-domain transfer learning strategy for tumor detection on brain MRI.. Diagnostics.

[125] Pandey S.K., Bhandari A.K. (2023). Morphological transfer learning based brain tumor detection using YOLOv5.. Multimedia Tools Appl..

[126] Ashafuddula M., Islam N.I., Islam R. (2024). ContourTL-Net: Contour-based transfer learning algorithm for early-stage brain tumor detection.. Int J Biomed Imaging.

[127] Rastogi D., Johri P., Kadry S., Kim S., Kumar L., Baghela V.S., Khan A.A. (2025). XcepFusion for brain tumor detection using a hybrid transfer learning framework with layer pruning and freezing.. Sci. Rep..

[128] Esteva A., Robicquet A., Ramsundar B., Kuleshov V., DePristo M., Chou K., Cui C., Corrado G., Thrun S., Dean J. (2019). A guide to deep learning in healthcare.. Nat Med.

[129] Yang Q., Liu Y., Chen T., Tong Y. (2019). Federated machine learning.. ACM Trans. Intell. Syst. Technol..

[130] Guan H., Liu M. (2022). Domain adaptation for medical image analysis: A survey.. IEEE Trans. Biomed. Eng..

[131] Yu X., Wang J., Hong Q.Q., Teku R., Wang S.H., Zhang Y.D. (2022). Transfer learning for medical images analyses: A survey.. Neurocomputing.

[132] Miller T. (2019). Explanation in artificial intelligence: Insights from the social sciences.. Artif Intell.

[133] Yosinski J., Clune J., Bengio Y., Lipson H. How transferable are features in deep neural networks?. NIPS'14: Proceedings of the 28th International Conference on Neural Information Processing Systems.

[134] Huang G., Liu Z., Van Der Maaten L., Weinberger K.Q. Densely connected convolutional networks.. 2017 IEEE Conference on Computer Vision and Pattern Recognition (CVPR).

[135] Shorten C., Khoshgoftaar T.M. (2019). A survey on image data augmentation for deep learning.. J. Big Data.

[136] Al-Saleh A., Tejani G.G., Mishra S., Sharma S.K., Mousavirad S.J. (2025). A federated learning-based privacy-preserving image processing framework for brain tumor detection from CT scans.. Sci. Rep..

[137] El Houby E.M. (2025). Using transfer learning for diabetic retinopathy stage classification.. Appl Comput Inform.

